# Programmable self-organization of heterogeneous microrobot collectives

**DOI:** 10.1073/pnas.2221913120

**Published:** 2023-06-05

**Authors:** Steven Ceron, Gaurav Gardi, Kirstin Petersen, Metin Sitti

**Affiliations:** ^a^Sibley School of Mechanical and Aerospace Engineering, Cornell University, Ithaca, NY 14853; ^b^Computer Science and Artificial Intelligence Lab, Massachusetts Institute of Technology, Cambridge, MA 02139; ^c^Physical Intelligence Department, Max Planck Institute for Intelligent Systems, Stuttgart 70569, Germany; ^d^Department of Physics, University of Stuttgart, Stuttgart 70569, Germany; ^e^Electrical and Computer Engineering, Cornell University, Ithaca, NY 14853; ^f^Institute for Biomedical Engineering, Eidgenössische Technische Hochschule Zurich, 8092 Zurich, Switzerland; ^g^School of Medicine and College of Engineering, Koç University, Istanbul 34450, Turkey

**Keywords:** microrobotics, self-organization, robot collectives, heterogeneity, programmable self-assembly

## Abstract

At the microscale, coupled physical interactions between collectives of agents can be exploited to enable self-organization. Past systems typically consist of identical agents; however, heterogeneous agents can exhibit asymmetric pairwise interactions which can be used to generate more diverse patterns of self-organization. Here, we study the effect of size heterogeneity in microrobot collectives composed of circular, magnetic microdisks on a fluid–air interface. Each microrobot spins or oscillates about its center axis in response to an external oscillating magnetic field, in turn producing local magnetic, hydrodynamic, and capillary forces that enable diverse collective behaviors. We demonstrate through physical experiments and simulations that the heterogeneous collective can exploit the differences in microrobot size to enable programmable self-organization, density, morphology, and interaction with external passive objects. Specifically, we can control the level of self-organization by microrobot size, enable organized aggregation, dispersion, and locomotion, change the overall shape of the collective from circular to ellipse, and cage or expel objects. We characterize the fundamental self-organization behavior across a parameter space of magnetic field frequency, relative disk size, and relative populations; we replicate the behavior through a physical model and a swarming coupled oscillator model to show that the dominant effect stems from asymmetric interactions between the different-sized disks. Our work furthers insights into self-organization in heterogeneous microrobot collectives and moves us closer to the goal of applying such collectives to programmable self-assembly and active matter.

## Significance

Microscale collectives composed of simple, locally reactive constituents can harness the effects of self-organization to enable diverse global behaviors. While phase separation of homogeneous collectives is well studied, heterogeneous collectives are relatively unexplored. This study focuses on a collective of magnetic microdisks of different sizes and examines how the group can self-organize into homogeneous subgroups using an external magnetic field. We find that heterogeneity enables collective behaviors including morphology reconfiguration, organized aggregation, dispersion, and locomotion, and caging and expulsion of external objects. Our work furthers insights into self-organization of heterogeneous microrobot collectives and may provide useful insights into the future of active matter.

Natural collectives at different length scales use physical attributes (1) or gene expressions (2–5) for self-organization. Inspired by these natural systems, scientists have been designing robot collectives at the macro and micro length scales to execute tasks like reconfiguration, locomotion, and manipulation (6–11). Often, it is beneficial for the collective’s constituents to exhibit heterogeneity to enable more complex behaviors that might otherwise not be possible through a homogeneous collective (12), like separation into various groups and task allocation. At the macroscale, onboard computation, sensing, and communication make heterogeneous pairwise interactions possible, which enable the collective to split into groups with different functionalities. At the microscale, however, similar actuation, sensing, and processing capabilities are not possible. Instead, heterogeneous interactions must stem from the physical forces acting between the microrobots and between the microrobots and their environment.

Here, we present an externally driven, heterogeneous microrobot collective in which we exploit reactive symmetric and asymmetric pairwise interactions for programmable self-organization to enable organized aggregation, dispersion, and locomotion, morphology reconfiguration, and object caging and expulsion through azimuthal flow fields. Our microrobots are circular ferromagnetic disks at the fluid–air interface that individually respond to an oscillating magnetic field. The microrobots interact via attractive magnetic dipole–dipole interactions and repulsive hydrodynamic forces, and the strength of the hydrodynamic force can be tuned by changing the frequency of the global magnetic field. The microrobots are heterogeneous by microdisk radius, with two to three discrete radii present in the system. A microrobot’s radius determines the attractive and repulsive forces it exerts on its surroundings, which enables the collective to transition back and forth between a disordered state (different-sized disks neighbor each other) and a self-organized state (same-sized disks neighbor each other). We study the self-organization behavior as a function of microrobot sizes and relative numbers and demonstrate how heterogeneity enables a microrobot collective to perform functions that are otherwise not possible through a homogeneous collective.

Past work on magnetic microrobot collectives has considered self-organization in systems with a homogeneous response to the external magnetic field (13–20) demonstrating self-organization into vortex-like formations (14, 17, 21, 22) and square-like grids (15) and on-demand reconfiguration between collective rotation, chains, and gas-like states that can be used to locomote, manipulate objects, and explore open areas (21). Magnetic microrobot collectives that operate by symmetry breaking on a submerged substrate also exhibit diverse behaviors and morphological reconfiguration (22). Although these systems consist of disks similar to ours, they focus on a homogeneous collective’s ability to change shape and function. Here, we advance the field several steps further: we demonstrate tunable self-organization in a heterogeneous collective where the pairwise forces between disks of different sizes are asymmetric; we demonstrate that these collectives can aggregate, disperse, and locomote while remaining organized by size; we show that the collective deforms anisotropically under isotropic compressive forces; and finally, we exploit the microrobots’ size heterogeneity to control large and small passive objects’ radial positions with respect to the collective centroid. With the exception of dispersion, all of these behaviors occur when the microrobots respond identically to the global magnetic field (all the microrobots oscillate identically to the external magnetic field). Organized dispersion is possible because of the heterogeneous response of the smaller microrobots to the global magnetic field (i.e. smaller microrobots oscillate asynchronously to the global magnetic field, while larger ones oscillate synchronously).

At the macroscale, physical self-organization has been studied in a heterogeneous collective composed of spinning gear-like rotors on a vibrating plate both via experiments (23, 24) and numerical simulations (25, 26); the collective separates by chirality through local physical interactions. Similar outcomes have been modeled for microscale systems of spinning gear-like motors with far-field hydrodynamic interactions (27), and colloidal spinners have been experimentally studied that can separate into distinct clusters (28). Very small collections of spinning magnetic particles have also been demonstrated to separate mostly by size (29). While also composed of spinning agents, our heterogeneous microrobot collectives are composed of many magnetic microdisks that span two to three different sizes, spin in the same direction, and interact with other disks near and far in the system via different physical interactions.

To complement our experimental work, we replicate self-organization through a physics-based (physical) model and a swarming coupled oscillator (swarmalator) model (30, 31). While the physical model does not quantitatively replicate experimental results due to the difficulty of tuning parameters, especially related to environmental conditions, it explains the detailed physical interactions between disks. To complement it, we also present the swarmalator model which replicates the disordered and self-organization behavior by using the dominating attractive and repulsive pairwise interactions while abstracting away detailed physics. This abstract framework summarizes the emergent behaviors as dependent on the disks’ radii and the magnetic field frequency and is useful for characterizing diverse real-world collective behaviors at the macro- and microscales since each agent’s state in that framework can represent both internal and physical attributes.

Our heterogeneous microrobot collective demonstrates the ability to switch between disordered and ordered states; this is the bedrock of self-assembly (32) and programmable matter (33), and thus, our system may lend itself to fundamental studies on self-organization (34). We study the disordered and self-organized states to determine how the degree of self-organization by microrobot size can be programmed through various system parameters. The results presented in this study have implications on both fundamental and application-oriented aspects of microrobotics; we hope that this work will accelerate the development of programmable matter (35–39) where the local mechanical properties are a function of the magnetic and hydrodynamic interactions between neighboring disks and can be altered by a single external field.

## Results

### System Overview.

Our microrobot collectives are tested within circular arenas with a concave air–water interface that increases the disk–boundary repulsion and drives the disks to coalesce at the center. Pairwise coupling among the disks is summarized by two pairwise interactions: the attractive magnetic dipole–dipole interactions and repulsive hydrodynamic forces. These interactions are modulated by the size and response of the disks to the external global magnetic field and are further discussed in *SI Appendix*, section S1. Graphical representations of the experimental setup, global field, and pairwise interactions are shown in *SI Appendix*, Figs. S1 and S2.

**Fig. 1.**
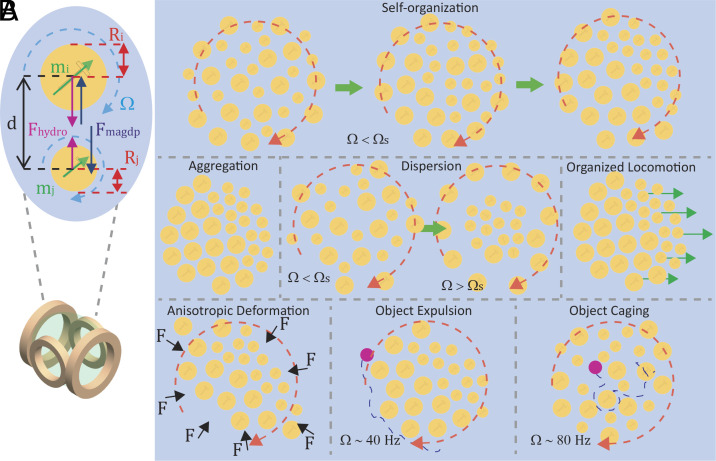
Heterogeneous microrobot collectives. (*A*) Schematic of the Helmholtz coil setup and the attractive magnetic dipole–dipole (angle averaged) and repulsive hydrodynamic interactions between two microrobots of different sizes. The magnitude of the attractive and repulsive forces that each disk exerts on its neighbors is dependent on its radius $R_{i,j}$ (red arrows), magnetic moment $m_{i,j}$ (green arrows), the frequencies of the external magnetic field $\Omega_{x,y}$ which controls how fast the microrobots spin or oscillate about their center axes (blue), and the distance from other microrobots ( d ). The pairwise interactions are asymmetric because the forces exerted by the larger microrobot are higher than those exerted by the smaller microrobot, as depicted by the purple and black arrows. (*B*) Overview of the various behaviors and functions possible with a heterogeneous microrobot collective. The collective can switch on demand between a self-organized state and a disordered state, exhibit organized aggregation, dispersal (the rotating magnetic field frequency *Ω* is higher than the smaller disks’ step-out frequency *Ω_s_*), and locomotion, deform anisotropically under isotropic compression, and cage or expel passive objects (purple) according to their size and the magnetic field frequency.

The collective behaviors are enabled by an external magnetic field produced by two pairs of perpendicular Helmholtz coils (graphically shown in Fig. 1*A*) that modulate their axial frequencies (Ω*_x_* and Ω*_y_*) to change the magnetic field vector (**B**) orientation within the workspace as:

[1] $$\begin{array}{c} B\left(t\right)=\left(B_{x}\cos(\Omega_{x}t)\right)∙\hat{x}+\left(B_{y}\sin(\Omega_{y}t)\right)∙\hat{y} \end{array},$$

where *B_x_* and *B_y_* are the axial field amplitudes (10 mT), and *t* is time. To limit the parameter space of this study, *B_x_* and *B_y_* remain equal and constant across all experiments. *Ω_x_* and *Ω_y_* are varied between 30 and 100 Hz, and *Ω_x_* = *Ω_y_* (= *Ω*) enables self-organization by microdisk size and exhibits a circular magnetic field profile. The circular magnetic field profile enables all the disks to spin about their respective center axes. Spinning disks create an azimuthal flow field around their respective perimeters and therefore collectively create large circular flow fields around the collective. Past works on homogeneous collectives have shown that a circular magnetic field can be used to create rotating collectives at the fluid–air and fluid–substrate interface (15, 22). An example of a homogeneous rotating collective is shown in Movie S1. The hydrodynamic force (*F_hydro_*) and the angle-averaged magnetic dipole–dipole (*F_magdp_*) force (see *SI Appendix*, sections S1 and S2 for more details) exerted by disk j on disk i along their center–center axis [*F^ij^_hydro_*(*d*) and *F^ij^_magdp_*(*d*)] depend on their sizes as:

[2] $$F_{\mathrm{hydro}}^{\mathrm{ij}}\left(d\right)=\frac{\rho R_{i}^{4}R_{j}^{3}\omega_{j}^{2}}{d^{3}},$$

[3] $$F_{\mathrm{magdp}}^{\mathrm{ij}}\left(d\right)=-\frac{3\mu_{0}m_{j}m_{i}}{4\pi d^{4}},$$

[4] $$m_{i}=\rho_{m}\pi R_{i}^{2},$$

Here, ρ is the density of water, *R_i_* and *R_j_* are the radius of the *i^th^* and *j^th^* disks, respectively, *ω_j_* is the angular velocity of disk *j*, *m_i_* and *m_j_* are the magnetic moments of the *i^th^* and *j^th^* disks, respectively, *ρ_m_* is the magnetic moment per unit area and is estimated to be ~ 0.1 A for a cobalt thin film of thickness 500 nm, and *d* is the center-to-center distance between the two disks.

In homogeneous microrobot collectives, where *R_i_* = *R_j_* across all pairwise interactions, all the agents exert identical forces on each other; however, in collectives containing different-sized disks, a larger disk exerts a higher repulsion on a smaller disk than vice versa. More information on these asymmetric pairwise interactions can be found in *SI Appendix*, section S3. Owing to the inequality in forces, the rotating collectives present in this work differ from their homogeneous counterparts in their local organization. For most of this study, we focus on the circular magnetic field profile to study a variety of self-organized behaviors at different magnetic field frequencies. Later, we introduce a second linear relationship between the axial magnetic field frequencies to transition the collective to a static mode where all agents individually oscillate about their own axis, which drives the collective to cease rotation and instead aggregate while remaining in a self-organized state.

### Heterogeneity and Pairwise Interactions.

The main pairwise interaction forces between disks are the attractive magnetic dipole–dipole force and the repulsive hydrodynamic force; the various parameters that determine the strength of these forces are depicted in Fig. 1*A*. A microrobot’s response to the global magnetic field is enabled by the amount of magnetic material onboard. The disks in this study are fabricated simultaneously but are heterogeneous by radius (further described in *Materials and Methods*); therefore, all have the same thickness of magnetic material, but those with a larger radius have a higher magnetic material volume and thus a higher net magnetic moment. The higher magnetic moment enables larger disks to have a higher step-out frequency, which means that at sufficiently high frequencies, part of the collective can be stepped out (small disks stop following the external magnetic field), while the remainder continues to exhibit continuous rotation. Disks with a larger radius exhibit higher hydrodynamic repulsion on neighboring agents (Eq. **2**); this force scales with a disk’s radius to the third power and its instantaneous angular velocity to the second power, which means that even a slight increase in disk size can significantly increase repulsion. An intricate balance between the hydrodynamic repulsion and the magnetic dipole–dipole attraction determines neighbor spacing, which affects collective self-organization at a specific frequency range.

The combination of the two pairwise interaction forces enables the collective to exhibit different behaviors at different magnetic field frequencies. Through our studies, we find that there is a particular magnetic field frequency range that enables the collective to self-organize by disk size. Examples of heterogeneous collectives that transition from mixed to separated groups in physical experiments are shown in Movies S2–S7. If the magnetic field frequency is too low (*Ω* < 15 Hz), the repulsive hydrodynamic forces are not large enough to overcome the attractive magnetic dipole–dipole forces. If the frequency is high, but below the step-out frequency of the smaller disks (~60 to 80 Hz), disorder persists since disks are strongly repelled, and this inhibits self-organization. If the step-out frequency affects only part of the collective (~80 to 100 Hz), this enables another form of self-organization. The ordered and disordered regions of the magnetic field frequency parameter space are discussed in the following.

### Self-Organization by Microrobot Size.

Self-organization is studied for two cases when there are two disk sizes in the collective: 1) when Ω is below all the disks’ step-out frequencies, and 2) when *Ω* is above the smaller disks’ step-out frequency (*Ω_s_*). Self-organization for case 1) is shown in physical experiments, through simulations that model the physical interactions between the disks, and through an abstract model that reduces the system to a collective of mobile coupled oscillators. For case 2), we present experimental results to highlight the emergent behaviors when some constituents have stepped out; these behaviors are not modeled because the instantaneous angular velocities of the stepped-out disks no longer remain continuous, and therefore, their physical interactions are not replicated by our models. We encourage the reader to refer to the *SI Appendix* and Movies S1–S15 to better understand each of the emergent behaviors.

Self-organization is quantified through an order parameter that measures how well the collective has spatially separated by disk size. The separation order parameter (*Z_s_*), which holds a value between 0 and 1, is defined as

[5] $$Z_{s}=\frac{1}{N}\sum_{i=1}^{N}\frac{S_{i}}{n_{i}}.$$

where *N* is the total number of disks in the collective, *n_i_* is the total number of Voronoi neighbors of disk *i*, and *S_i_* is the number of disk *i*’s Voronoi neighbors with a similar radius. *Z_s_* = 1 if there are no disks with a Voronoi neighbor that has a different radius than itself, which implies that the collective is homogeneous. With heterogeneity, even collectives that completely separate into two groups of large and small disks may hold a separation order parameter value between 0.5 and 1 because of the boundary regions between the two groups.

When *Ω* < *Ω_s_*, agents increase their neighbor spacing with increasing *Ω* because of the higher hydrodynamic repulsion resulting from a faster spin speed. This leads to frequency ranges in which the collective spatially separates by disk size. The level of separation is measured across the parameter spaces *Ω* – *N_12_* and *Ω* – A*_12_*, where *N*_12_ = *N*_1_/*N*_2_ is the ratio of the number of larger disks (*N*_1_) to number of smaller disks (*N*_2_), and *A_12_ = A_1_/A_2_* is the ratio of the total area of large disks to small disks. The parameter space is tested for three pairs of disk sizes: 1) *R*_1_ = 200 μm, *R*_2_ = 125 μm, 2) *R*_1_ = 200 μm, *R*_2_ = 150 μm, and 3) *R*_1_ = 175 μm, *R*_2_ = 150 μm. In each set of tests, the number of disks with *R*_1_ is held constant, while those with *R*_2_ are increased so that *A_12_* ∈ [0.6, 2.0] across the three pairs of disk sizes; the resulting *N_12_* ranges are *N_12_* ∈ [0.23, 0.78] for case 1), *N_12_* ∈ [0.34, 1.1] for case 2), and *N_12_* ∈ [0.43, 1.46] for case 3).

Snapshots of the self-organization when *Ω* < *Ω_s_* are shown for various *Ω* and *A*_12_ for the different combinations of radii in *SI Appendix*, Figs. S3–S5 and Movies S4–S6.

**Fig. 2.**
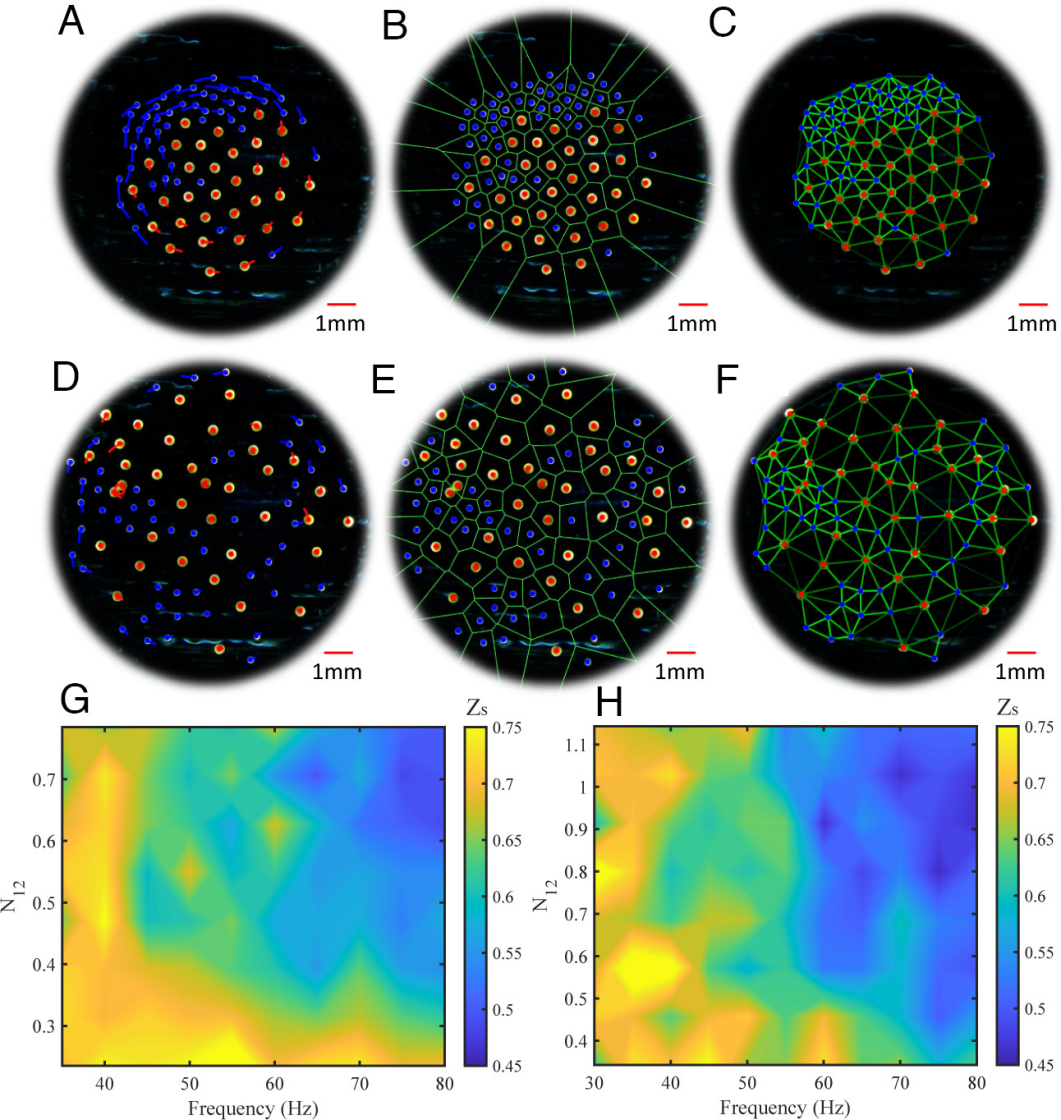
Self-organization by microrobot size. (*A*–*C*) Experimental images for *R*_1_ = 200 μm and *R*_2_ = 125 μm, *N*_12_ = 0.63, and *Ω* = 40 Hz. (*D*–*F*) Experimental images for *R*_1_ = 200 μm and *R*_2_ = 125 μm, *N*_12_ = 0.71, and *Ω* = 80 Hz. (*A* and *D*) Show the trajectories of large (red) and small (blue) disks. The polygons in *B* and *E* show the Voronoi tessellation. (*C* and *F*) Show the lines connecting Voronoi neighbors; the lines in each experiment are dark green if the disks are far apart and bright green if closer together. The lines are drawn using image processing for better visualization. The red and blue circles in *A*–*F* represent the centers of the large and small disks, respectively. (*G* and *H*) Heat map of the separation order parameter $Z_{s}$ across the parameter space of magnetic field frequency and number ratio of disks (*Ω* – *N*_12_) for (*G*) *R*_1_ = 200 μm and R_2_ = 125 μm and (*H*) *R*_1_ = 200 μm and *R*_2_ = 150 μm. The bright yellow regions in the heat maps indicate the sections of the parameter space in which there is the highest separation of large and small disks.

Collectives with *R*_1_ = 200 μm and *R*_2_ = 125 μm, shown in Figs. 2 *A* and *B*, demonstrate the best separation by size since there is a large difference between the repulsive forces of disks with *R*_1_ and *R*_2_; the number of disks with *R*_2_ is held constant at 51, while the number of disks with *R*_1_ is increased from 12 to 40; note that the change in the collective’s size due to addition of disks negligibly increases the collective–boundary interactions since the collective’s area increase is very small in comparison to the area of the arena. Fig. 2*A* shows an example when there is a total of 83 disks (*N_12_* = 0.63) and *Ω* = 40 Hz; this highlights the separation behavior of small disks (blue) and larger disks (red) by showing their trajectories (Fig. 2*A*), the Voronoi tessellation (Fig. 2*B*), and the connections between Voronoi neighbors (Fig. 2*C*).

As shown in Figs. 2 *A*–*C*, the small disks tend to be pushed toward the edge of the collective. This results from the asymmetric hydrodynamic interactions between the large and small disks; large disks exert higher hydrodynamic repulsion on the smaller disks than vice versa (as can also be seen from Eq. **2**). Additionally, each rotating disk produces an azimuthal flow, and the strength of that flow also depends on the size of the disk generating it. Therefore, the larger disks generate stronger azimuthal flows than the smaller disks since the strength of the flow is proportional to a disk’s radius cubed. When another disk or passive object is present near a rotating disk, it is dragged by the azimuthal flow and starts moving tangential to the line crossing between the centers of the two members. This causes the whole collective to orbit around a common center of mass. Once the disks have separated by the sizes (large disks at the center and the small disks at the edges), the local direction of the net flow generated by the small and the large disks is opposite to each other at the interface between the two groups. Especially when there are larger differences in disk sizes, the flows produced by large disks dominate, and therefore, the small disks tend to spread throughout the edges. These cases of self-organization demonstrate radial separation by size instead of lateral and are especially evident when the number of smaller disks is higher.

The flip side to this self-organized behavior is shown in Fig. 2*D* (*N_12_* = 0.71 and *Ω* = 80 Hz) where there is high disorder and no noticeable difference (from the trajectories) in the disks’ angular velocities. As shown in Movie S2, a heterogeneous collective can transition from a disordered state (*Ω* = 80 Hz) to a self-organized state (*Ω* = 40 Hz) and back to a disordered state.

Figs. 2 *E* and *F* show that there is wide and narrow spacing between neighboring disks across the collective; this is caused by small and large disks being Voronoi neighbors throughout all regions. Fig. 2 *G* and *H* show *Z*_s_ across the *N*_12_ – *Ω* parameter space when the magnetic field frequency is below all disks’ step-out frequency. Fig. 2*G* corresponds to collectives with *R*_1_ = 200 μm and *R*_2_ = 125 μm and shows that the self-organization by disk size can be achieved across all *Ω* at low *N*_12_ and across all *N*_12_ at low *Ω*. At high *Ω* and *N*_12_, disks exert higher repulsion and generate large azimuthal flow fields that cause disks to disrupt their neighbors’ motions. The same general trends are displayed by collectives with *R*_1_ = 200 μm and *R*_2_ = 150 μm; however, there are patches of lower *Z*_s_ that can be attributed to the low difference between repulsive forces generated by the different sizes of disks. A low difference in the repulsive forces means that the pairwise forces between two disks with differing radii will be very similar, which will lead to more mixing. This is shown in *SI Appendix*, Figs. S6 and S7, where the pairwise forces for collectives of different disk size combinations are plotted as a function of pairwise distance and magnetic field frequency. Collectives with *R*_1_ = 200 μm and *R*_2_ = 125 μm have the largest difference between pairwise forces and thus the best separation by disk size; collectives with *R*_1_ = 175 μm and *R*_2_ = 150 μm have little difference in the pairwise forces and thus almost no separation by disk size.

The deformation of a collective under external forces would depend on the neighbor spacing and the local pairwise interactions between the microrobots (*SI Appendix*, section S3). We may consider the collective as a network of springs, in which there is a spring between all microrobots near to each other; however, a more in-depth analysis is beyond the scope of this work and is reserved for a future study. In contrast to homogeneous collectives, heterogeneous collectives can possess spatially varying neighbor spacing (*SI Appendix*, Fig. S8) and asymmetric pairwise interactions, and we demonstrate in later sections how these two factors can be exploited to enable anisotropic morphology reconfiguration in heterogeneous collectives as compared to isotropic reconfiguration that is expected of homogeneous collectives.

More information on each of these collectives’ self-organization and additional characterizations across the *A*_12_ – *Ω* parameter space can be found in *SI Appendix*, Figs. S9–S14. Collectives with even larger differences between disk sizes are shown in Movie S6; here, there are 25 disks with a radius of 200 μm and 100 disks with a radius of 50 μm. Similar size separation is observed; however, the small disks step out at a low magnetic field frequency (~35 Hz), which causes the collective to appear highly disordered from 40 to 80 Hz. At low magnetic field frequencies, like 20 and 30 Hz, the small disks are pushed toward the edges of the collective, while the large disks rotate closer to the collective’s center. Furthermore, the small disks exert very low repulsive forces on each other, and they are unable to overcome any attractive magnetic dipole–dipole forces; this causes many of them to remain attached throughout the experiments.

Similar self-organization is observed in collectives with three disk sizes, as shown in Movie S7. These collectives have *R*_1_ = 200 μm, *R*_2_ = 125 μm, and *R*_3_ = 50 μm; the smallest disks tend to crowd around the mid-sized disks, and the largest disks tend to remain toward the center of the collective. The main driver of size separation in these collectives is the repulsive interaction between the largest disks and the mid-sized disks; this radially organizes the collective into regions of large disks and small disks. Since the smallest disks are more strongly repelled by the larger disks than by the mid-sized disks, they tend to distribute themselves throughout the regions occupied by mid-sized disks.

### Organized, Static Aggregation.

**Fig. 3.**
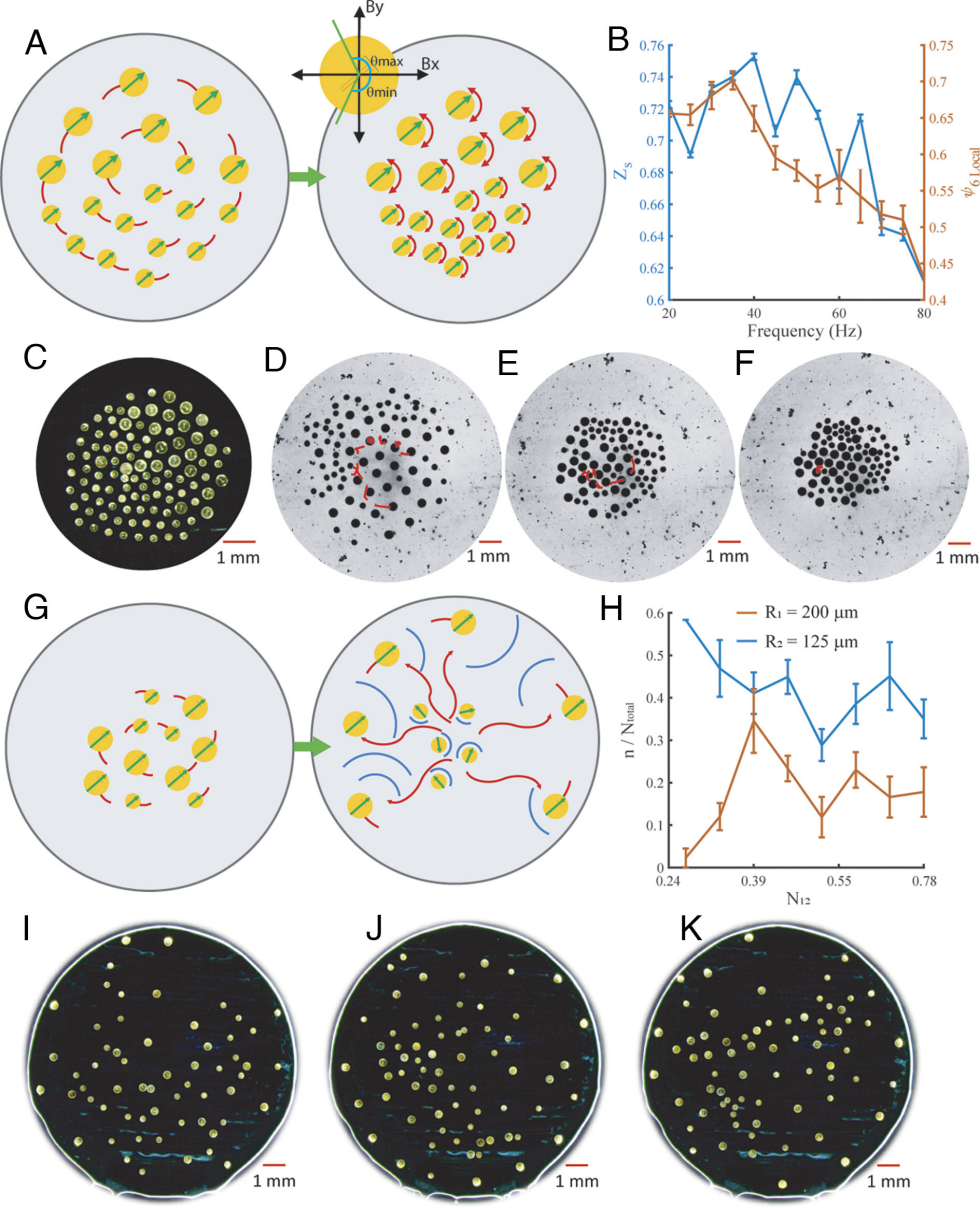
Organized aggregation and dispersion. (*A*) A rotating magnetic field vector creates a self-organized rotating collective with high hydrodynamic repulsion between neighbors; the oscillating magnetic field vector can maintain a static, ordered collective with disks oscillating about their own center axis between *θ*_min_ and *θ*_max_, which generates much lower hydrodynamic repulsion between neighboring disks. (*B*) Separation (*Z*_s_) and local hexatic ( $\psi_{6_{\mathrm{Local}}}$ ) order parameters for static collectives; averages and error bars are across 10 s of video. (*C*) Aggregated collective organized by microrobot size after transitioning to static mode with *Ω*_x_ = 60 Hz, *Ω*_y_ = 30 Hz. (*D*–*F*) Passive tracer particle (radius ~ 2 - 3 μm) trajectory when the collective exhibits rotation mode and static mode (Movie S9). (*D*) Trajectory during 2.7 s when collective is rotating. (*E*) Trajectory during the next 5.7 s when the collective has transitioned to static mode and is shrinking in radius. (*F*) Trajectory during the next 8.3 s which is after the collective has aggregated to a stable radius. (*G*) At higher frequencies, the smaller disks step out and repel less, while larger disks continue following the magnetic field vector and repel each other strongly, which enables larger microrobots to move toward the arena boundary. This enables the collective to disperse while remaining organized by size. (*H*) Fraction of large (*R*_1_ = 200 μm) and small (*R*_2_ = 125 μm) disks at a distance from the collective center at or above 0.7× the collective radius; the *y *axis is *n/N_total_*, where *n* is *N_1_* (number of large disks) or *N_2_* (number of small disks), and *N_total_* is *N_1_* + *N_2_*; averages and error bars are across 10 s of video (*Ω* = 100 Hz). (*I*–*K*) Experiment snapshots of collectives driven at 100 Hz with *R*_1_ = 200 μm and *R*_2_ = 125 μm at (*I*) *N*_12_ = 0.63, (*J*) *N*_12_ = 0.71, and (*K*) *N*_12_ = 0.78.

Up to now, all self-organization by size has been maintained within rotating collectives; however, Fig. 3 and Movies S8 and S9 show that the collective can aggregate and maintain its organized state after it transitions to a static mode. This is achieved by applying the following linear relationship between the two axial magnetic field frequencies: *Ω_x_* = 2*Ω_y_*. Instead of keeping the disks rotating at a constant angular velocity about their own axes, the signal drives them to oscillate between *θ*_min_ and *θ*_max_ (Fig. 3*A*) and cancel the azimuthal flow fields around them, which enables the collective to remain in a globally static state. In each of the experiments shown, the collective rotates and is self-organized by disk size and then transitions to the static mode. As shown in Fig. 3*B*, the collective can maintain a high separation order throughout the range of 20 to 80 Hz; however, because of the axial frequency relationship *Ω_y_* = 2*Ω_x_*_,_ *Ω*_y_ quickly surpasses the smaller disks’ step-out frequency which leads to lower repulsion and clustering. The clustering causes a noticeable reduction in the local hexatic order (Fig. 3*B*); the hexatic order parameter calculations are shown in Eqs. **9** and **10** in the *Materials and Methods* section. At lower frequencies (20 to 30 Hz), the static mode generates less repulsion between agents than the rotation mode, which means that neighboring disks significantly decrease their pairwise distance and exhibit similar spacing throughout the whole collective (Fig. 3*C*); this is shown in all snapshots in Figs. 3 *D*–*F*, demonstrating how the collective shrinks from an expanded, rotating group to an aggregated, static state. In Figs. 3 *D*–*F*, an aggregation of tracer particles (each with radius ~ 2 to 3 μm) was tracked as they flowed across the collective throughout the transition from the rotation mode to static mode. During the rotation mode, the tracer particles traverse a large spatial region during 2.7 s because of the strong and variable azimuthal flow fields (Fig. 3*D*); however, when the magnetic field switches to static mode, the tracer particles move a smaller amount in a window of 5.7 s (Fig. 3*E*). The decrease in motion is caused by the oscillating azimuthal flow fields which become smaller as the collective transitions to an aggregated state. After the collective has transitioned to the aggregated state, the tracer particles are almost stationary as they oscillate about the perimeter of a single disk (Fig. 3*F*); the low neighbor spacing in the aggregated state prohibits large azimuthal flow fields that can drive the tracer particles to move between microrobots.

### Organized Dispersion in the Step-Out Regime.

At higher magnetic field frequencies (*Ω* > 80 Hz), smaller disks begin to step out since they hold a lower amount of magnetic material than larger disks; this enables the collective to disperse with the large disks moving toward the boundary. Although the disks tend to separate by size, the emergent self-organization is entirely different than the one shown earlier because the smaller disks no longer follow a rotating magnetic field vector with an angular velocity equal to that of the magnetic field.

The organized dispersion is shown in Fig. 3 *G*–*K* and Movie S10; at 100 Hz, large disks tend to move toward the edges of the arena boundary, which leaves most smaller disks closer to the arena center. This self-organization could be attributed to 1) the smaller disks ceasing to repel each other strongly once they have stepped out and are rotating about their axis at a slower angular velocity and 2) the larger disks continuing to repel each other strongly and maximizing their distance from any other disks by going to the arena boundary; this transition to the step-out regime is shown in Fig. 3*G*. The fraction of large and small disks at a distance greater than 0.7x the collective radius is shown in Fig. 3*H*, where the fraction of larger disks is always higher than the smaller disks because of the higher repulsion they generate, which leads them to the edge of the collective. The behavior is shown in experimental snapshots for various *N*_12_ in Figs. 3 *I*–*K*. There is a distribution of step-out frequencies for disks of each size, and if the sizes of two disks are closer, the distributions may overlap, and the distinction is unclear between the behavior of the disks if they are too similar in size; this is the case for collectives with *R*_1_ = 200 μm, *R*_2_ = 150 μm and *R*_1_ = 175 μm, *R*_2_ = 150 μm.

### Physical and Swarmalator Models.

**Fig. 4.**
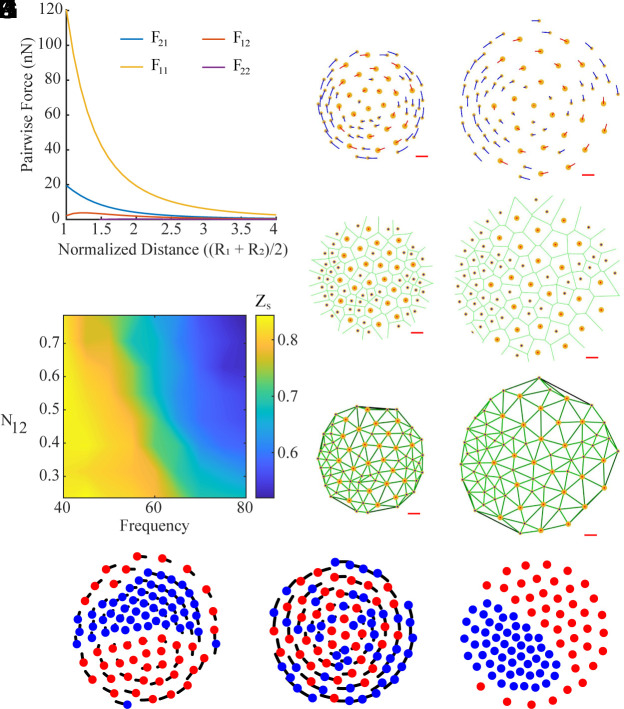
Modeling heterogeneous microrobot collectives. (*A*) Pairwise interaction forces as a function of normalized distance between two microrobots of sizes *R*_1_ = 200 μm and *R*_2_ = 125 μm, when *Ω* = 40 Hz. *F*_21_ is the force exerted from a large microrobot on a small microrobot, *F*_12_ is the force exerted from a small microrobot on a large microrobot, *F*_11_ is the force exerted by a large microrobot on another large microrobot, and *F*_22_ is the force exerted by a small microrobot on another small microrobot. (*B*–*D*) Physical model simulation of collectives with *R*_1_ = 200 μm and *R*_2_ = 125 μm, *N*_12_ = 0.47, and *Ω* = 40 Hz. (*E*–*G*) Physical model simulation when *R*_1_ = 200 μm and *R*_2_ = 125 μm, *N*_12_ = 0.47, and *Ω* = 80 Hz. (*B* and *E*) Show the trajectories of simulated disks. (*C* and *F*) show the voronoi separation of simulated collectives. (*D* and *G*) Show the lines connecting Voronoi neighbors in each simulation. The lines are dark green if the disks are far apart and bright green if closer together. Red scale bars in *A*–*F* correspond to 1 mm. (*H*) Heat map of the separation order parameter *Z*_s_ across the parameter space of magnetic field frequency and number ratio of disks (*Ω* – *N*_12_). (*I* and *J*) Swarmalator trajectories when collectives are in rotation mode (*I* and *J*) and static mode (*K*); parameters are *R*_1_ = 200 μm, *R*_2_ = 125 μm, *N*_12_ = 0.78, and *Ω* = 40 Hz (*I*), *N*_12_ = 0.78 and *Ω* = 80 Hz (*J*), and *N*_12_ = 0.78, and *Ω_x_* = 80 Hz, *Ω_y_* = 40 Hz (*K*).

We use two models to simulate the self-organization observed in experiments (Fig. 4). The first model uses the physical interactions between the disks; the physical model introduced by Wang et al. (15) has been adapted to replicate the asymmetric interactions between disks of different sizes. An example of pairwise forces between a pair of disks when R_1_ = 200 μm and R_2_ = 125 μm is shown in Fig. 4*A*; it can be seen from the plot that smaller disks exert weaker forces on each other as compared to the bigger disks and the force exerted by a small disk on a big disk is weaker than vice versa. The large difference in the forces between pairs of disks with R_1_ and R_1_ vs. pairs of disks with R_2_ and R_2_ allows the collective to self-organize more easily when the difference between R_1_ and R_2_ is larger. As shown in *SI Appendix*, Fig. S6, the difference between the four combinations of pairwise forces decreases when R_1_ and R_2_ have similar values, like R_1_ = 175 μm and R_2_ = 150 μm; this results in lower self-organization in those cases. *SI Appendix*, section A.2, defines the physical model which describes the disks’ pairwise interactions among themselves, the arena boundary, and the effect of the water curvature against the arena boundary. The physical model simulations (shown in Movie S11) demonstrate qualitative similarities with the experiments (Fig. 4 *B*–*G*) and a separation order comparable to that observed in the experiments; however, a clear demarcation between the self-organized and disordered regions, as observed in experiments, is missing in the simulation results, as shown in *SI Appendix*, Fig. S15. This could be caused by a slight mismatch in the estimated force due to the curved air–water interface that is used for the simulations (see *Materials and Methods* and *SI Appendix*, section A.2 for more details on the curvature force calculations).

The second model strips away the physical forces and instead models the pairwise interactions as attractive and repulsive forces between swarming coupled oscillators that are modeled as point particles, also commonly referred to as swarmalators (30, 31). In the physical model, the hydrodynamic repulsion and magnetic dipole–dipole interactions between disks depend on the disks’ radii. Larger disks exert larger repulsive and attractive forces than the smaller disks; as a result, to simply explore the self-organization of the collective, while ignoring the actual physical interactions between disks, the behaviors are summarized as dependent on the disks’ radii and the magnetic field frequency. Each swarmalator is a point particle (its radius is zero) and represents a disk in the physical system; the oscillator phase represents the corresponding disk’s radius. The coupling between any two swarmalators depends on their phases and the external magnetic field frequency.

The swarmalator model is described in more detail in the *Materials and Methods* and a graphical representation of the pairwise swarmalator interactions is shown in *SI Appendix*, Fig. S16. All disks are subject to the confinement boundary effects of the circular arena and aggregate at the center when the magnetic field is turned off; this can be summarized by equal spatial attraction among all agents. In this model, disks with a similar phase attract, and those with differing phases repel. Coupled oscillators and swarmalators are usually defined by phases having values between 0 and 2π; in this case, a disk’s radius acts as the oscillator phase. In the physical system, the disks do not change their radius over time; therefore, the swarmalator model assumes that all agents have a natural frequency of zero, and there is no change in the phase. The model was tuned through neighbor spacing, which is dependent on agents’ radii, and the separation order parameter, which depends on the magnetic field frequency. Agents with a smaller radius have lower spacing with Voronoi neighbors than those with larger radii. A lower magnetic field frequency tends to result in a higher separation order than a higher frequency. These properties are consistent with the physical system’s behavior and were controlled through several parameters explained in more detail in the *Materials and Methods* section.

Fig. 4*H* demonstrates a similar self-organization to the experiments across *N*_12_ – *Ω* parameter space in collectives with *R*_1_ = 200 μm and *R*_2_ = 125 μm. In Fig. 4 *I*–*K* and Movie S12, the swarmalator model exhibits similar separation behavior to the physical experiments when the collective is self-organized and disordered. In other words, the physical interactions can be ignored, and instead, the separation behavior can be summarized by two parameters, disk radius and magnetic field frequency.

### Forces Exerted on a Heterogeneous Collective.

When the collective self-organizes by size, the asymmetric pairwise interactions across the collective enable regional differences in cohesion. This means that there will be a different collective response to external forces across the group that depends on each microrobot’s size and its pairwise interactions with neighboring microrobots. We consider two types of external forces acting on our microrobot collective; the first is an isotropic compressive force that is more or less equal on all sides of the collective and points toward the group’s center, and the second type of force is applied in a single direction.

**Fig. 5.**
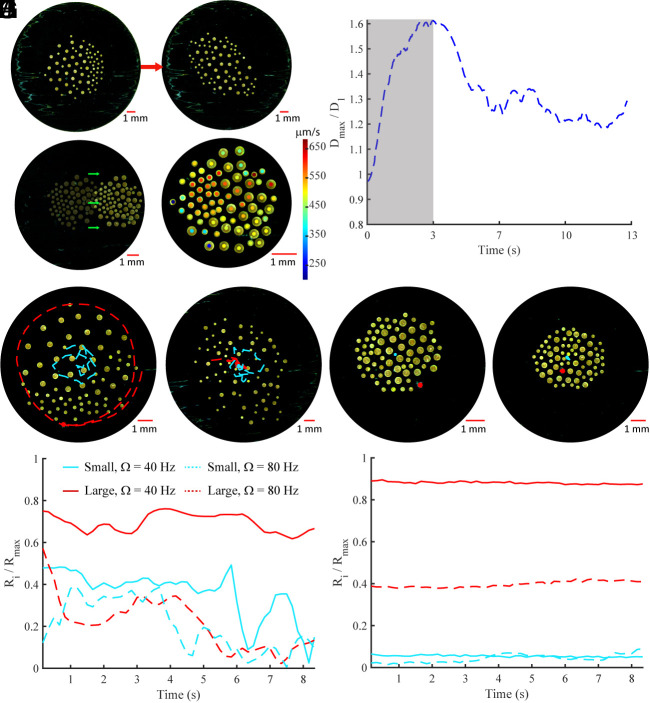
Forces exerted on and by a heterogeneous collective. (*A*) Self-organized collectives deform anisotropically when compressed by isotropic boundary repulsion forces. (*B*) Major axis length of a heterogeneous collective in the rotation mode when it is compressed by boundary forces; the gray region represents the transient period of external disturbances. (*C*) A static collective remains organized by microrobot size while translating when a magnetic field gradient is applied in the positive x-direction. (*A* and *C*) *Ω_x_* = 60 Hz and *Ω_y_* = 30 Hz. (*D*) Velocity in the x-direction for each microrobot in the locomoting collective shown in *C*. (*E*) Rotating collective that is self-organized by microrobot size keeps large passive particles on the periphery and encapsulates small passive particles (*Ω* = 40 Hz). (*F*) Rotating collective that is disorganized by microrobot size brings the large particle to its center (*Ω* = 80 Hz). Heterogeneous collectives maintain the position of passive particles after transitioning to static mode: (*G* and *H*) *Ω_x_* = 60 Hz and *Ω_y_* = 30 Hz. *R*_1_ = 200 μm, *R*_2_ = 125 μm, and *N*_12_ = 0.63 in *A* and *C*–*H*. (*I*) Radial position of large and small passive particles, with respect to the collective centroid, corresponding to the experiments in *E* and *F*. (*J*) Radial position of large and small passive particles corresponding to the experiments in *G* and *H*.

In the first case, the collective experiences an isotropic compressive force because of an increase in the arena boundary repulsion force. The experiments are conducted in a circular arena with the water slightly lower than the circular enclosure’s height so that a small meniscus forms which produces weak repulsion that makes the collective gravitate toward the center of the arena. If the water level is lowered, the meniscus becomes larger which increases the boundary repulsion and acts as an isotropic compressive force on the microrobot collective. In our experiments, this compressive force resulted from 1 mL of water removed from the arena. Although it is difficult to determine the exact force since we do not know the exact shape of the meniscus, we know that the compressive force is more or less isotropic and enough to overcome the pairwise interactions between microrobots to enable compression of the collective. A homogeneous collective experiencing this axisymmetric force will deform isotropically since all pairwise interactions are symmetric. A heterogeneous collective, however, has varying levels of cohesion across the group, which enables it to deform anisotropically. As shown in Fig. 5*A*, the collective passively reconfigures its morphology from a circular formation to an ellipse; the microrobots remain self-organized by size but shift their position about the center of the collective until they reach a steady-state configuration after the external forces have been applied. Fig. 5*B* shows how the length of the major axis of a rotating collective fluctuates after the isotropic compressive force has been applied. After the transient period caused by the water removal, the collective settles in an ellipse-like configuration with a higher major axis length.

The second type of external force is achieved through a magnetic field gradient, which drives the aggregated collective (Fig. 5*C*) to move in a specified direction. In a homogeneous collective, all microrobots move at the same relative speed because of the equal spacing between constituents, but in a heterogeneous collective, the constituents move at variable velocities, as shown in Fig. 5*D*. Higher spacing between large disks at the boundary of the collective makes them move slower, whereas the smaller disks exhibit lower repulsion on their neighbors causing denser clusters, which respond more strongly under a magnetic field gradient causing those clusters to move faster. The behaviors explored for collectives experiencing isotropic compressive forces and directional forces were further explored through several rotating and aggregated collectives and are shown in Movies S13 and S14.

### Forces Exerted by a Heterogeneous Collective.

Beyond the ability to passively reconfigure morphology and maintain an organized state in response to external forces, the heterogeneous collective may also use the forces generated by the collective’s azimuthal flow fields to cage or expel passive objects. As shown in Fig. 5 *E*–*H*, a collective with the parameters *R*_1_ = 200 μm, *R*_2_ = 125 μm, and *N*_12_ = 0.63 can exploit the asymmetric pairwise interactions to control the radial positions of two passive objects of radii of 20 μm and 200 μm, with respect to the collective’s centroid. When a rotating field is set to *Ω* = 40 Hz (Fig. 5*E*), the group self-organizes by size which enables different sections of the collective to have variable azimuthal flow fields. Larger microrobots create larger azimuthal flow fields which enable higher repulsion that expel large passive objects to the boundary. Smaller passive objects are not repelled as strongly given their smaller size and instead become trapped in the azimuthal flows at the collective’s center. When the frequency is *Ω* = 80 Hz (Fig. 5*F*), such that the collective becomes disordered, the larger azimuthal flow fields are no longer concentrated at the edge of the collective; this enables the passive objects to be pulled toward the center of the group. By changing the magnetic field profile to the static mode, the collective aggregates and causes the passive objects to maintain a static position, whereas the azimuthal flow fields make the larger passive object remain at the boundary (*Ω* = 40 Hz) or stay caged in the center (*Ω* = 80 Hz).

As shown in Fig. 5*I*, the radial positions of the large and small passive objects fluctuate within a range over time when the collective is rotating. This is caused by the variable azimuthal flow fields that enable the passive objects to move about the perimeter of various microrobots; this is especially the case when the passive objects are caged at the collective’s center. When the collective is static and aggregates, however, the passive objects maintain a specific position (Fig. 5*J*). It is worth noting that the transition between the different behaviors shown here is possible because of the careful selection of passive object sizes given the sizes of the microrobots. If the objects had radii comparable to the larger microrobots, the larger objects would have gone to the collective’s edge at low and high frequencies; this occurs because the hydrodynamic repulsion increases with passive particle size which pushes the larger object outward. As shown in Movie S15, the expulsion behavior persists at low and high frequencies when two large passive objects aggregate to form an object larger than the large microrobots; the three small passive objects remain caged. All of the object caging and expulsion behaviors are shown in Movie S15.

## Discussion

In summary, we present a microrobot collective that is heterogeneous by disk size and is capable of programmable self-organization based on one global magnetic field. We study the self-organization by varying 1) the magnetic field frequency, 2) the magnetic field profile, 3) the number of disks in the collective, 4) the number ratio of larger disks to smaller disks, and 5) the relative size of the disks. Collectives composed of disks with radii of 200 μm and 125 μm separate into different regions. Collectives with radii that have a lower difference in size, like those composed of disks with radii of 200 μm and 150 μm or 175 μm and 150 μm exhibit lower degrees of self-organization. We also demonstrate that after self-organizing a collective through a circular magnetic field profile (a rotating signal), the collective can aggregate and retain its organized state even when it transitions to a parabolic field profile (a static collective). At higher rotation frequencies (*Ω* = 100 Hz), the collective can disperse through a self-organized state in which the small disks (radius of 125 μm) step out and remain closer to the center of the collective, while many of the larger disks do not step out and exert high hydrodynamic repulsion which pushes them to the outer edge of the collective.

We further highlight that the heterogeneous collectives behave differently to their homogeneous counterparts when an external force is applied to them. We show that the application of an isotropic force on a heterogeneous collective causes anisotropic deformation of the collective, which could be attributed to the asymmetric pairwise interactions and thus the spatially varying cohesion within the heterogeneous collectives. We also show that the collective can retain its self-organized state, even though it is performing locomotion under a directional magnetic gradient force.

Additionally, we show that the dissimilar azimuthal flows produced by large and small microdisks in the rotating collectives can be used to cage and expel passive objects of different sizes. At smaller frequencies (*Ω* = 40 Hz), bigger passive objects are expelled to the edge of the collective, and the smaller objects are caged within the bulk. On switching to higher frequencies (*Ω* = 80 Hz), both the bigger and smaller objects are caged within the bulk of the collective.

Finally, we demonstrate that the general separation behavior achieved with a rotating magnetic field signal can be qualitatively reproduced through a physical model and a swarmalator model. We found that while the physical model reproduces the general separation behavior well, the demarcation between the two regions of order and disorder is unclear, likely because of the curved fluid-air interface which is not reproduced well in the model. The swarmalator model reproduces the general self-organization (both in rotating and static modes) and can be tuned to agree with the experiments’ general range of the separation order parameter throughout the *N*_12_ – *Ω* parameter space. The swarmalator model abstracts away the system-specific physical interactions and uses general attraction and repulsion terms to mimic a real-world collective behavior. Therefore, it could be used to predict states in other microscale systems with dynamic heterogeneity and various physical interactions, like particle size changing over time through acoustic stimuli or chemical reactions (38).

Robot collectives are advantageous in resource-constrained systems because they can support complex behaviors beyond the sum of their parts. This is especially true at the microscale, where the individual robot functionality is not an effect of explicitly programmed sensor-action behaviors, but rather an effect of local physical (magnetic, hydrodynamic, and capillary) interactions from many robots superimposed on their shared environment. By introducing heterogeneous collectives in this paper, we demonstrated an unprecedented variety of behaviors enabled by asymmetric pairwise interactions between robots, including on-demand self-organization by size, organized aggregation, dispersion, and locomotion, morphology reconfiguration, and caging and expulsion of passive objects. We envision that our study will be valuable to researchers across domains concerned with heterogeneous groups of interacting agents and will impact work ranging from the development of microrobot collectives useful for microscale assembly to the improved understanding and characterization of behaviors in naturally occurring heterogeneous collectives.

## Materials and Methods

### Fabrication of the Microdisks.

The microdisks are printed using two-photon polymerization-based 3D microprinting (Nanoscribe Photonic Professional GT), and nanofilms of 500 nm cobalt and 60 nm gold are then sputtered onto them using Kurt J. Lesker NANO 36.

### Video Acquisition and Analysis.

The experiments are performed under a Leica manual zoom microscope Z16 APO, and they are recorded using a Basler acA2500-60uc camera. A LED light source SugarCUBE Ultra illuminator connected to a ring light guide (0.83" ID, Edmund Optics #54-176) is used to illuminate the microrobots. A Python script was developed using the OpenCV library to process the experimental videos and extract the positions of the microrobots. The analysis of the processed data was done using MATLAB. The raw images were used without any enhancements for the processing.

### Experimental Protocol.

The experiments are carried out in a 15-mm-diameter circular arena. The air–water interface is adjusted to be slightly concave. Each self-organization test is preceded by disordering the collective first at a high magnetic field frequency between 80 and 100 Hz and then switching to the lower frequency being tested for that specific case.

### Simulations.

The numerical model developed by Wang et al. (15) is modified to simulate microdisks with different radii. In the simulations, the initial positions of the microdisks are chosen to be randomly sampled from a uniform distribution and ensuring no overlaps between any two microdisks. See *SI Appendix*, sections A.1 and A.2, for details on the physical model.

### Calculation of Curvature Force.

The curvature force is modeled as an effective attraction to the center of the arena. It is nontrivial to measure the effect of curvature precisely because the curvature could change slowly during the experiments due to the evaporation of water. Therefore, multiple numerical simulations are performed using different values for the coefficient of the curvature force such that it fits the average neighbor distance within the collective for different magnetic field frequencies and number ratios (*N*_12_). See *SI Appendix*, section A.2, for more details.

### Swarmalator Model.

The swarmalator model is defined by the equation of motion as

[6] $$\begin{array}{c} {\dot{x}}_{i}\left(t\right)=\frac{1}{N}\overset{N}{\underset{j\neq i}{\sum }}A\frac{x_{j}-x_{i}}{\left|x_{j}-x_{i}\right|}-\frac{x_{j}-x_{i}}{{\left|x_{j}-x_{i}\right|}^{2}}\left(B+J\cos\left(\frac{\pi }{2}\left(3+\frac{\left|\theta_{j}-\theta_{i}\right|}{\theta_{j}+\theta_{i}}\right)\right)\right)\\ \;-\frac{\theta_{j}}{\theta_{max}}\frac{\left(x_{j}-x_{i}\right)}{{\left|x_{j}-x_{i}\right|}^{3}}-\frac{c\theta_{j}}{\theta_{max}}\frac{\left(x_{j}-x_{i}\right)}{{\left|x_{j}-x_{i}\right|}^{3}}\times \;\hat{z}, \end{array}$$

[7] $$\theta_{i}=R_{i}^{2},$$

[8] $$J=\frac{\Omega_{s}-\Omega }{\left(\Omega_{s}-\Omega_{\min}\right)},$$

where N is the number of microdisks in the collective, ***x****_i_* is the position of the *i^th^* swarmalator, and *A* and *B* are the normalized coefficients of global attraction and repulsion terms, respectively; they both have a value of 1. The phase, *θ_i_*, is dependent on the radius (*R_i_*) of the disk corresponding to the *i^th^* swarmalator. *Ω* is the magnetic field frequency, *Ω_s_* is the step-out frequency for the small microdisks (~ 80 Hz), and *Ω_min_* is the frequency at which clustering occurs (~ 20 Hz). Throughout the simulations, *Ω* is modulated between 40 and 80 Hz to switch the *J* coefficient between 0 and 1. *J* determines how much agents with similar *θ* will spatially attract to each other; this enables the swarmalator model to replicate the general self-organization by size observed in the physical experiments. When *J* is closer to 1, agents with a similar *θ* tend to attract more strongly which enables the collective to self-organize by phase; when J is closer to zero, there is low attraction between agents with a similar phase which enables the swarmalators to replicate the mixing behavior from the physical experiments. As shown in the physical experiments, self-organization by size happens when the magnetic field frequency is lower (~20 to 50 Hz); therefore, Eq. **8** is designed so that *J* is higher at lower magnetic field frequencies. The neighbor spacing was further tuned by *θ_j_* which is normalized in the equation of motion by the largest *θ* value in the collective, *θ*_max_. Agents with the lower *θ* value will repel other agents less which enables the collective to reproduce the lower spacing between agents with a lower radius. Finally, a cross-product term at the end of the equation of motion enables agents to rotate about each other; the *c* parameter can be switched to 1 to enable a rotating collective or zero to enable a static collective. This enables us to study the motion of the microrobot collective during rotation and static modes, even though the specific pairwise physical interactions have been abstracted away.

### Calculation of the Hexatic Order Parameter.

The local hexatic order parameter is calculated as:

[9] $$\psi_{6_{\mathrm{Local}}}=\frac{\Sigma_{k}\left|e^{i6\theta_{k}}\right|}{K},$$

where *K* is the total number of neighbors of all microdisks, *k* is the neighbor index, and *θ_k_* is the polar angle of the vector from each microdisk to the *k^th^* neighbor. The global hexatic order parameter is calculated as:


[10] $$\psi_{6_{\mathrm{Global}}}=\left|\frac{\Sigma_{k}e^{i6\theta_{k}}}{K}\right|.$$

### Calculation of the Hydrodynamic Lift Force.

The hydrodynamic lift force on a disk is exerted along the perpendicular to the local direction of the shear flow (12, 39).

[11] $$F_{\mathrm{hydro}}^{\mathrm{ij}}\left(d\right)\propto \mu R_{i}v_{i}Re_{G},$$

[12] $$v_{i}=\omega_{j}R_{i},$$

[13] $$Re_{G}=\frac{\rho R_{1}^{2}G}{\mu },$$

[14] $$G\propto \frac{\omega_{j}R_{j}^{3}}{d^{3}},$$

[15] $$\begin{array}{c} F_{\mathrm{hydro}}^{\mathrm{ij}}\left(d\right)\propto \mu R_{i}\left(\omega_{j}R_{i}\right)\left(\frac{\rho R_{i}^{2}}{\mu }\cdot \frac{\omega_{j}R_{j}^{3}}{d^{3}}\right)=c_{\mathrm{hydro}}\cdot \frac{\rho R_{i}^{4}R_{j}^{3}\omega_{j}^{2}}{d^{3}} \end{array}.$$

where μ is the dynamic viscosity of water (10^−3^ Pa·s), $R_{i}$ is the radius of the $i^{\mathrm{th}}$ microdisk, $v_{i}$ is the typical velocity of the $i^{\mathrm{th}}$ disk in the flow created by the $j^{\mathrm{th}}$ disk, Re is the Reynolds number, ρ is the density of water (10^3^ kg/m^3^), and G is the shear rate at the location of the $i^{\mathrm{th}}$ disk generated due to the rotation of the $j^{\mathrm{th}}$ disk. The proportionality constant $c_{\mathrm{hydro}}$ is empirically found to be 1 for our system, therefore resulting in Eq. **2**.

## Supplementary Material

Appendix 01 (PDF)

Movie S1. Homogeneous collective *R* = 125 *μ*m.

Movie S2. Heterogeneous Collectives Transition Between Order and Disorder.

Movie S3. Heterogeneous Collectives (*R*_1_ = 200 *μ*m, *R*_2_ = 125 *μ*m).

Movie S4. Heterogeneous Collectives (*R*_1_ = 200 *μ*m, *R*_2_ = 150 *μ*m).

Movie S5. Heterogeneous Collectives (*R*_1_ = 175 *μ*m, *R*_2_ = 150 *μ*m).

Movie S6. Heterogeneous Collectives (*R*_1_ = 200 *μ*m, *R*_2_ = 50 *μ*m) *N*_R__1_ = 25, *N*_R__2_ = 100.

Movie S7. Heterogeneous Collectives (*R*_1_ = 200 *μ*m, *R*_2_ = 125 *μ*m, *R*_3_ = 50 *μ*m).

Movie S8. Organized Static Aggregation.

Movie S9. Flow Visualization.

Movie S10. Organized Dispersal at 100 Hz.

Movie S11. Physical Model Simulations of Heterogeneous Collectives (*R*_1_ = 200 *μ*m, *R*_2_ = 125 *μ*m).

Movie S12. Swarmalator Model Simulations of Heterogeneous Collectives (*R*_1_ = 200 *μ*m, *R*_2_ = 125 *μ*m).

Movie S13. Anisotropic Deformation Under Isotropic Compression.

Movie S14. Organized Collective Locomotion.

Movie S15. Caging and Expulsion of Passive Objects.

## Data Availability

All data needed to evaluate the conclusions of this study are present in the paper and its *SI Appendix*, Supplementary Information Files. Data is also available on Zenodo (https://zenodo.org/record/7803838#.ZC4AXnbMJ3g) (40). All study data are included in the article and/or supporting information.
