# Supplementary material for: Programmable self-organization of heterogeneous microrobot collectives
Source: Proc Natl Acad Sci U S A. 2023 Jun 5;120(24):e2221913120. doi: 10.1073/pnas.2221913120 (PMC10268276; doi:10.1073/pnas.2221913120)
Supplement: Supplementary file 1 — Appendix 01 (PDF) [file pnas.2221913120.sapp.pdf]

PNAS

Supporting Information for
Programmable Self-Organization of Heterogeneous Microrobot Collectives

Steven Ceron[1,2†], Gaurav Gardi[3,4†], Kirstin Petersen[5*], Metin Sitti[3,6,7*]

[1] Sibley School of Mechanical and Aerospace Engineering, Cornell University; Ithaca, NY 14853, USA.
[2] Computer Science and Artificial Intelligence Lab, Massachusetts Institute of Technology; Cambridge, MA 02139, USA.
[3] Physical Intelligence Department, Max Planck Institute for Intelligent Systems; 70569, Stuttgart, Germany.
[4] Department of Physics, University of Stuttgart, 70569 Stuttgart, Germany
[5] Electrical and Computer Engineering, Cornell University; Ithaca, NY 14853, USA
[6] Institute for Biomedical Engineering, ETH Zurich, 8092 Zurich, Switzerland
[7] School of Medicine and College of Engineering, Koç University, 34450 Istanbul, Turkey

[†] These authors contributed equally.
* Metin Sitti and Kirstin Petersen
Email: sitti@is.mpg.de, kirstin@cornell.edu

This PDF file includes:

Appendix A

Section 1. Pairwise Interaction Forces.
Section 2. Physical Model.
Section 3. Asymmetric Pairwise Interactions.
Section 4. Calculation of entropy by neighbor distances.
Fig. S1. Experimental setup.
Fig. S2. Global field and pairwise interactions.
Fig. S3. Emergent self-organization of collectives with $R_1 = 200\ \mu$m, $R_1 = 125\ \mu$m.
Fig. S4. Emergent self-organization of collectives with $R_1 = 200\ \mu$m, $R_1 = 150\ \mu$m.
Fig. S5. Emergent self-organization of collectives with $R_1 = 175\ \mu$m, $R_1 = 150\ \mu$m.
Fig. S6. Pairwise Interactions.
Fig. S7. Frequency-Dependent Pairwise Interactions 3D Maps
Fig. S8. Average neighbor distance 3D plot.
Fig. S9. Characterization of collectives with $R_1 = 200\ \mu$m, $R_1 = 125\ \mu$m.
Fig. S10. Characterization of collectives with $R_1 = 200\ \mu$m, $R_1 = 150\ \mu$m.
Fig. S11. Characterization of collectives with $R_1 = 175\ \mu$m, $R_1 = 150\ \mu$m.
Fig. S12. Self-organization and entropy of collectives with $R_1 = 200\ \mu$m, $R_1 = 125\ \mu$m.
Fig. S13. Self-organization and entropy of collectives with $R_1 = 200\ \mu$m, $R_1 = 150\ \mu$m.
Fig. S14. Self-organization and entropy of collectives with $R_1 = 175\ \mu$m, $R_1 = 150\ \mu$m.
Fig. S15. Physical model characterization.
Fig. S16. Swarmalator model overview.

Other supporting materials for this manuscript include the following:

Movie S1. Homogeneous collective $R = 125\ \mu$m.
Movie S2. Heterogeneous Collectives Transition Between Order and Disorder.
Movie S3. Heterogeneous Collectives ($R_1 = 200\ \mu$m, $R_2 = 125\ \mu$m).
Movie S4. Heterogeneous Collectives ($R_1 = 200\ \mu$m, $R_2 = 150\ \mu$m).
Movie S5. Heterogeneous Collectives ($R_1 = 175\ \mu$m, $R_2 = 150\ \mu$m).
Movie S6. Heterogeneous Collectives ($R_1 = 200\ \mu$m, $R_2 = 50\ \mu$m) $N_{R_1} = 25, N_{R_2} = 100$.
Movie S7. Heterogeneous Collectives ($R_1 = 200\ \mu$m, $R_2 = 125\ \mu$m, $R_3 = 50\ \mu$m).
Movie S8. Organized Static Aggregation.
Movie S9. Flow Visualization.
Movie S10. Organized Dispersal at 100 Hz.
Movie S11. Physical Model Simulations of Heterogeneous Collectives ($R_1 = 200\ \mu$m, $R_2 = 125\ \mu$m).
Movie S12. Swarmalator Model Simulations of Heterogeneous Collectives ($R_1 = 200\ \mu$m, $R_2 = 125\ \mu$m).
Movie S13. Anisotropic Deformation Under Isotropic Compression.
Movie S14. Organized Collective Locomotion.
Movie S15. Caging and Expulsion of Passive Objects.

Section 1. Pairwise Interaction Forces

This discussion reviews the pairwise interactions present in our microrobot collective system: the magnetic dipole-dipole force, the capillary force, and the hydrodynamic force.

Magnetic dipole force: Each micro-disk is sputtered with a Cobalt thin film that make the disks ferromagnetic with a permanent magnetic dipole. The magnetic dipole-dipole force is attractive on average over one rotation and is caused by the magnetic dipole on each micro-disk. A rotating magnetic field exerts magnetic torque on each micro-disk; this causes each micro-disk's dipole to align with the instantaneous magnetic field vector and spin about its own axis. At high frequencies, in the step-out regime, micro-disks are unable to spin at the magnetic field vector's angular velocity; here, a micro-disk's response to the magnetic field is more erratic and in the right situations can lead to interesting emergent behaviors. The step-out frequency is dependent on several parameters, including the volume of the magnetic material on each micro-disk, the fluid medium's viscosity, and the magnitude of the external magnetic field.

Capillary force: Capillary interactions are weak since the micro-disks are flat; however, Supplementary Fig. 1 shows a flat 400 μm - diameter micro-disk at a fluid interface where small irregularities around its perimeter slightly deform the air-water interface; this enables weak capillary forces along those regions. Previous studies with a similar system show that micro-disks' edges can be patterned with corrugations to increase capillary attraction along certain orientations to form various lattice structures; two micro-disks experience attractive capillary forces when their corrugations are aligned and are repulsive when they are misaligned; at lower magnetic field frequencies, these micro-disks' capillary interactions dominate, and they are able to aggregate into specific types of structures. For the purposes of this study, micro-disks have no edge corrugations, and the numerical model assumes that there are no capillary interactions between agents.

Hydrodynamic force: The third pairwise interaction is the repulsive hydrodynamic force which is modulated by micro-disks' rotation behavior in response to the rotating magnetic field.

The hydrodynamic lift force is dependent on the size of the micro-disks and it can be non-symmetric if the sizes of the two micro-disks are different. The hydrodynamic force enables micro-disks to increase their neighbor distance; as the rotation frequency increases so does the repulsive force, which causes the collective to have a larger radius at a higher frequency. When the rotation frequency surpasses a micro-disk's step-out frequency, its angular velocity becomes non-uniform and the hydrodynamic repulsion it exerts on neighbors is significantly reduced. Throughout most experiments, we keep the rotation frequency below the micro-disks' step-out frequency which enables us to use a numerical model to reproduce some of the self-organization behaviors we observe in experiments. In the last portion of our experimental self-organization, we demonstrate that the collective exhibits a different form of self-organization when part of the collective has stepped out.

Section 2. Physical model.

The model used for simulations was adapted from literature[20] and modified to include the different magnetic field profile:

$$\frac{d\boldsymbol{r}_i}{dt} = \sum_{j\neq i}(6\pi\mu R_i)^{-1}\left(F_{mag-on,i,j}\left(r_{ji},\phi_{ji}\right) + \frac{\rho\omega_j^2 R_i^4 R_j^3}{r_{ji}{}^2}\right)\cdot\hat{r}_{ji} + \sum_{j\neq i}\left(\frac{F_{mag-off,i,j}\left(r_{ji},\phi_{ji}\right)}{6\pi\mu R_i} - \frac{R_j^3\omega_j}{r_{ji}{}^2}\right)\cdot\hat{r}_{ji}\times\hat{z} + \frac{\rho\omega_i{}^2 R_i^7}{6\pi\mu R_i}\cdot\left(\left(\frac{1}{d_{toLeft}{}^3} - \frac{1}{d_{toRight}{}^3}\right)\cdot\hat{x} + \left(\frac{1}{d_{toBottom}{}^3} - \frac{1}{d_{toTop}{}^3}\right)\cdot\hat{y}\right) + \frac{F_{curvature}}{6\pi\mu R_i}\cdot\frac{\mathbf{r}_{\text{center}} - \mathbf{r}_{\text{i}}}{R_{arena}}, \quad i = 1, 2, \ldots \tag{1}$$

$$\frac{d\alpha_i}{dt} = \frac{m_i B\sin(\theta-\alpha_i)}{8\pi\mu R_i^3} + \sum_{j\neq i}\frac{T_{mag-d,i,j}\left(r_{ji},\phi_{ji}\right)}{8\pi\mu R_i^3}, \quad i = 1,2,\ldots \tag{2}$$

$$\mathbf{B}(t) = \left(B_{x_0}\cos(\Omega_x t) + B_{x_1}\right)\cdot\hat{x} + \left(B_{y_0}\cos\left(\Omega_y t\right) + B_{y_1}\right)\cdot\hat{y}\ , \tag{3}$$

where r_i and r_j are the position vectors of micro-disks;
$r_{ji} = r_i - r_j$ is the vector pointing from the center of micro-disk j to the center of micro-disks i;
α_i and α_j are the orientations of micro-disks;
ϕ_{ji} is the angle of dipole moment with respect to r_{ji}. It is assumed to be the same for both micro-disks, as $\phi_{ji} = \phi_i = \phi_j$;
ω_i is the instantaneous spin speed of i^{th}micro-disk;
$B = |\mathbf{B}|$ is the magnetic field strength (10 mT);
$\theta = \arctan(B_y/B_x)$ is the orientation of the external magnetic field;
Ω_x and Ω_y are the oscillation frequencies of the x and y component of the external magnetic field respectively;
R_i is the radius of ith micro-disk;
μ is the dynamic viscosity of water (10^{-3} Pa·s);
ρ is the density of water (10^3 kg/m^3);
m_i is the magnetic dipole moment of the ith micro-disk ($0.44 * R_i^2$ A·m^2);
$F_{mag-on,i,j}$ and $F_{mag-off,i,j}$ are the magnetic dipole force on and off the center-to-center axis, respectively, and they are functions of r_{ji} and ϕ_{ji}; (see Ref. 20 or the details)
$T_{mag-d,i,j}$ is the magnetic dipole torque, and it is a function of r_{ji} and ϕ_{ji};
$F_{cap,i,j}$ is the capillary force, and it is a function of r_{ji} and ϕ_{ji} and embeds the symmetry of a micro-disk;
$T_{cap,i,j}$ is the capillary torque, and it is a function of r_{ji} and ϕ_{ji} and embeds the symmetry of a micro-disk;
d_{toLeft}, $d_{toRight}$, $d_{toBottom}$, and d_{toTop} are the distances of a micro-disk to the four sides of the physical boundary;
$F_{curvature}$ is the magnitude of the force due to curvature of the air-water interface and is set to be 1×10^{-9} N;
$\mathbf{r}_{\text{center}}$ is the position vector of the center of the arena;
R_{arena} is the radius of the arena.

If the center-center distance $r_{ji} <$ lubrication threshold ($2.1\times\frac{(R_i+R_j)}{2}$) and $r_{ji} \geq R_i + R_j$,

$$\mu \frac{d\boldsymbol{r_i}}{dt} = \sum_{j\neq i} A\left(\frac{d_{ji}}{R_i}\right)\left(F_{mag-on,\,i,j}\big(r_{ji},\varphi_{ji}\big) + \frac{\rho\omega_j^2 R_i^4 R_j^3}{{r_{ji}}^2}\right)\hat{r}_{ji}$$
$$+ \sum_{j\neq i} B\left(\frac{d_{ji}}{R_i}\right) F_{mag-off,\,i,j}\big(r_{ji},\varphi_{ji}\big)\,\hat{r}_{ji}\times\hat{\mathrm{z}}$$
$$+ \sum_{j\neq i} C\left(\frac{d_{ji}}{R_i}\right) mB\sin(\theta-\alpha_i)\,\hat{r}_{ji}\times\hat{\mathrm{z}}$$
$$+ \frac{\rho\omega_j^2 R_i^7}{R_i}\left(\left(\frac{1}{d_{toLeft}^3} - \frac{1}{d_{toRight}^3}\right)\hat{x} \right. \tag{4}$$
$$\left. + \left(\frac{1}{d_{toBottom}^3} - \frac{1}{d_{toTop}^3}\right)\hat{y}\right) + \frac{F_{curvature}}{6\pi\mu R_i}$$
$$\cdot \frac{\mathbf{r}_{\mathrm{center}} - \mathbf{r}_{\mathrm{i}}}{R_{arena}},\ i = 1,2,\ldots$$

$$\mu \frac{d\alpha_i}{dt} = G\left(\frac{d_{smallest}}{R_i}\right) mB\sin(\theta-\alpha_i)$$
$$+ \sum_{j\neq i} G\left(\frac{d_{ji}}{R_i}\right) T_{mag-d,i,j}\big(r_{ji},\ \varphi_{ji}\big),\ \ i = 1,2,\ldots \tag{5}$$

where the coefficients $A(x), B(x), C(x) and\ G(x)$ are lubrication coefficients.

If the center-center distance $r_{ji} < R_i + R_j$, a repulsion term is added to the force equation,

$$\mu \frac{d\boldsymbol{r_i}}{dt} = \sum_{j\neq i} A(\varepsilon)\left(F_{mag-on,\,i,j}\big(R_i+R_j,\varphi_{ji}\big) + \frac{\rho\omega_j^2 R_i^4 R_j^3}{{r_{ji}}^2}\right)\hat{r}_{ji}$$
$$+ \sum_{j\neq i} \frac{F_{wallRepulsion}}{6\pi R_i}\frac{-d_{ji}}{\left(\frac{R_i+R_j}{2}\right)}\hat{r}_{ji}$$
$$+ \sum_{j\neq i} B(\varepsilon) F_{mag-off,\,i,j}\big(R_i+R_j,\varphi_{ji}\big)\,\hat{r}_{ji}\times\hat{\mathrm{z}}$$
$$+ \sum_{j\neq i} C(\varepsilon)\, mB\sin(\theta-\alpha_i)\,\hat{r}_{ji}\times\hat{\mathrm{z}} \tag{6}$$
$$+ \frac{\rho\omega_j^2 R_i^7}{R_i}\left(\left(\frac{1}{d_{toLeft}^3} - \frac{1}{d_{toRight}^3}\right)\hat{x} \right.$$
$$\left. + \left(\frac{1}{d_{toBottom}^3} - \frac{1}{d_{toTop}^3}\right)\hat{y}\right) + \frac{F_{curvature}}{6\pi\mu R_i}$$
$$\cdot \frac{\mathbf{r}_{\mathrm{center}} - \mathbf{r}_{\mathrm{i}}}{R_{arena}},\ i = 1,2,\ldots$$

$$\mu \frac{d\alpha_i}{dt} = G(\varepsilon) mB\sin(\theta-\alpha_i) + \sum_{j\neq i} G(\varepsilon) T_{mag-d,i,j}\big(R_i+R_j,\ \varphi_{ji}\big), i$$
$$= 1,2,\ldots \tag{7}$$

where ε is a small number (10^{-10} µm/R); $F_{wallRepulsion}$ is set to be 10^{-7} N.

Section 3. Asymmetric Pairwise Interactions.

The following pairwise interactions, $F_{hydro}^{ij}(d)$ (repulsive hydrodynamic force) and $F_{magdp}^{ij}(d)$ (attractive angle-averaged magnetic dipole-dipole force) describe the force exerted by disk i on disk j.

ρ: Density of water ($1000\,\frac{kg}{m^3}$)
μ_0: Magnetic constant ($4\pi \times 10^{-7}\ \frac{H}{m}$)
ρ_i: Magnetic moment per unit area of disk i ($0.1\ A$)
R_i: Radius of disk i
R_j: Radius of disk j
ω_j: Angular velocity of disk j
d: Distance between the centers of disks i and j
m_i: Magnetic moment of disk i
m_j: Magnetic moment of disk j

$$F_{hydro}^{ij}(d) = \frac{\rho R_i^4 R_j^3 \omega_j^2}{d^3}$$

$$F_{magdp}^{ij}(d) = -\frac{3\mu_0 m_j m_i}{4\pi d^4}$$

$$m_i = \rho_m \pi R_i^2$$

$$F_{total}^{ij}(d) = F_{hydro}^{ij}(d) + F_{magdp}^{ij}(d)$$

In a heterogeneous collective with two radii present in the system (R_1 and R_2), there are four different possible sets of pairwise interactions between the micro-disks. A disk with radius R_1 exerting forces on a disk with radius R_2; a disk with R_2 exerting forces on a disk with R_1; a disk with R_1 exerting forces on a disk with R_1; a disk with R_2 exerting forces on a disk with R_2. Each of these pairwise interactions result in a different total force ($F_{total}^{ij}(d)$), which means that the exerted force will be unequal between two neighboring disks of differing radii and thus the calculated local spring constant ($k_{eff} = \frac{F_{total}^{ij}(d)}{d}$) will depend on which disk (i or j) is larger. A simple calculation with $R_1 = 200\ \mu$m and $R_2 = 125\ \mu$m reveals the difference between two cases:

Case (1): $R_i = R_1$, $R_j = R_2$, $d = 425\ \mu$m

$$k_{eff} = 2.45 \times 10^{-6}\,\frac{N}{m}$$

Case (2): $R_i = R_2$, $R_j = R_1$, $d = 425\ \mu$m

$$k_{eff} = 4.72 \times 10^{-6}\,\frac{N}{m}$$

Section 4. Calculation of entropy by neighbor distances.

The Shannon entropy by neighbor distances (H_{NDist}) was calculated as introduced in reference 20. Three different versions of H_{NDist} were calculated: only considering neighbor pairs with identical disk size (either R_1 or R_2) or considering neighbor pairs having non-identical disks (at least one disk with radius R_1 and one with radius R_2). Neighbors were identified using Voronoi tessellation.

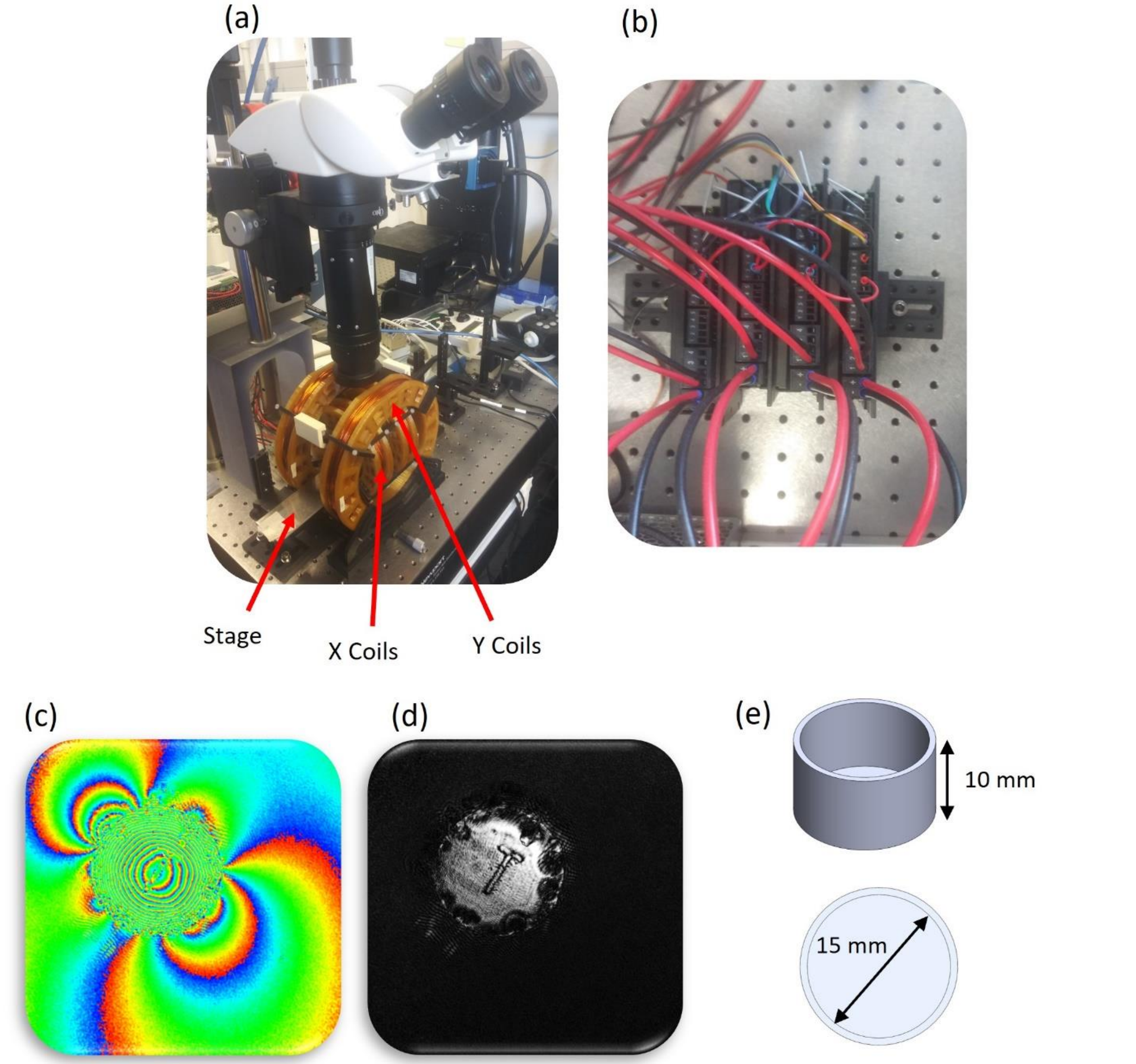

Fig. S1. Experimental setup. (a) Helmholtz coil setup. **(b)** The power supplies (Mean Well, SDR – 960 – 48) and motor servo controllers used as current controllers (Maxon ES-CON 70/10). **(c)** Phase map of 400 μm particle surface profile demonstrates that even with no corrugations, a particle can still exhibit some capillary forces on surrounding particles. **(d)** Intensity map of 400 μm particle. **(e)** Arena in which separation experiments were conducted.

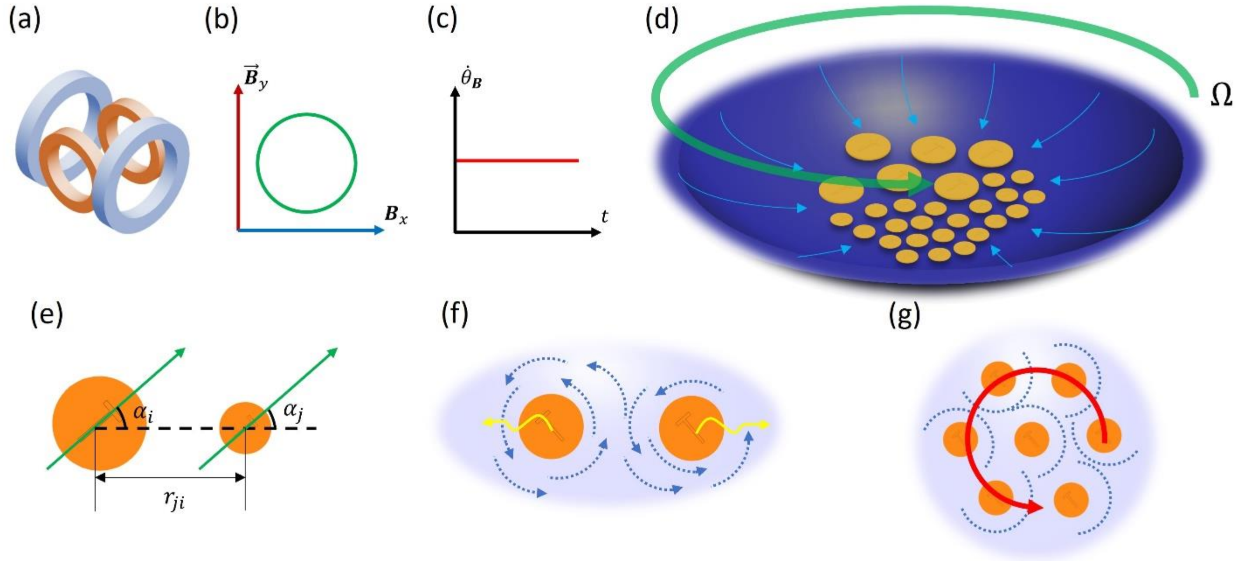

Fig. S2. Global field and pairwise interactions. (a) Magnetic coil set up with x and y coils colored in orange and blue, respectively (left). **(b)** Circular magnetic field profile used throughout the experiments (middle). **(c)** Magnetic field vector angular velocity remains constant with the rotating magnetic field. **(d)** Graphic representation of fluid-air interface where micro-disks coalesce towards the center of the circular because of their repulsive interaction with the boundary. The collective rotates because of the rotating magnetic field driven at a frequency Ω. **(e)** Each micro-disk aligns with the instantaneous magnetic field vector when the magnetic field frequency is below the step-out frequency and interacts with other micro-disks with a strong dependence on their respective distance. **(f)** As each micro-disk rotates about its center axis, it creates azimuthal flow fields and generates hydrodynamic repulsion that pushes it away from other micro-disks. **(g)** The combination of the azimuthal flow fields enables the collective to rotate about its common center of mass.

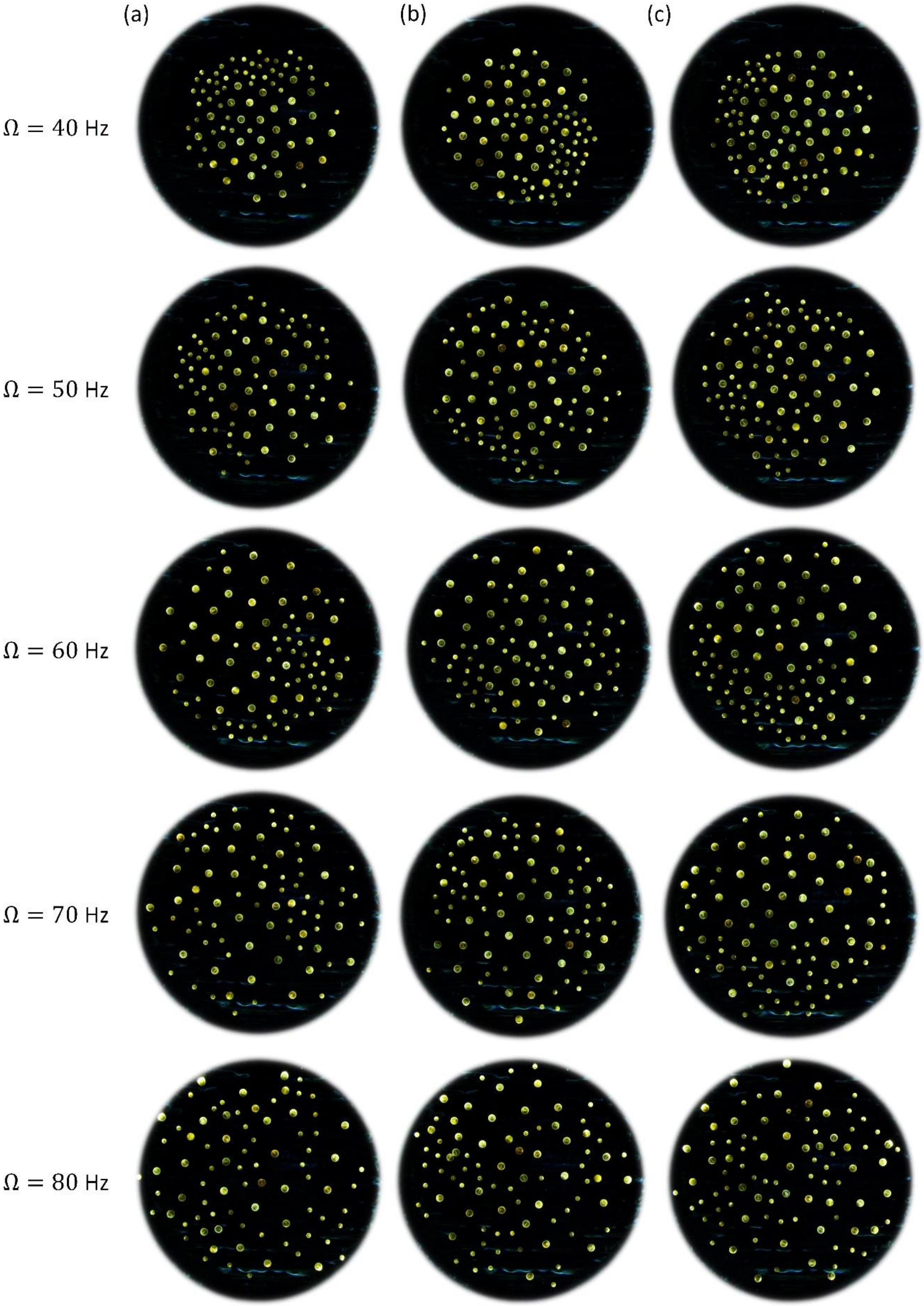

Fig. S3. Emergent self-organization of collectives with $\boldsymbol{R_1 = 200\ \mu}$m, $\boldsymbol{R_1 = 125\ \mu}$m. (a) $A_{12} = 1.6$. **(b)** $A_{12} = 1.8$. **(c)** $A_{12} = 2.0$.

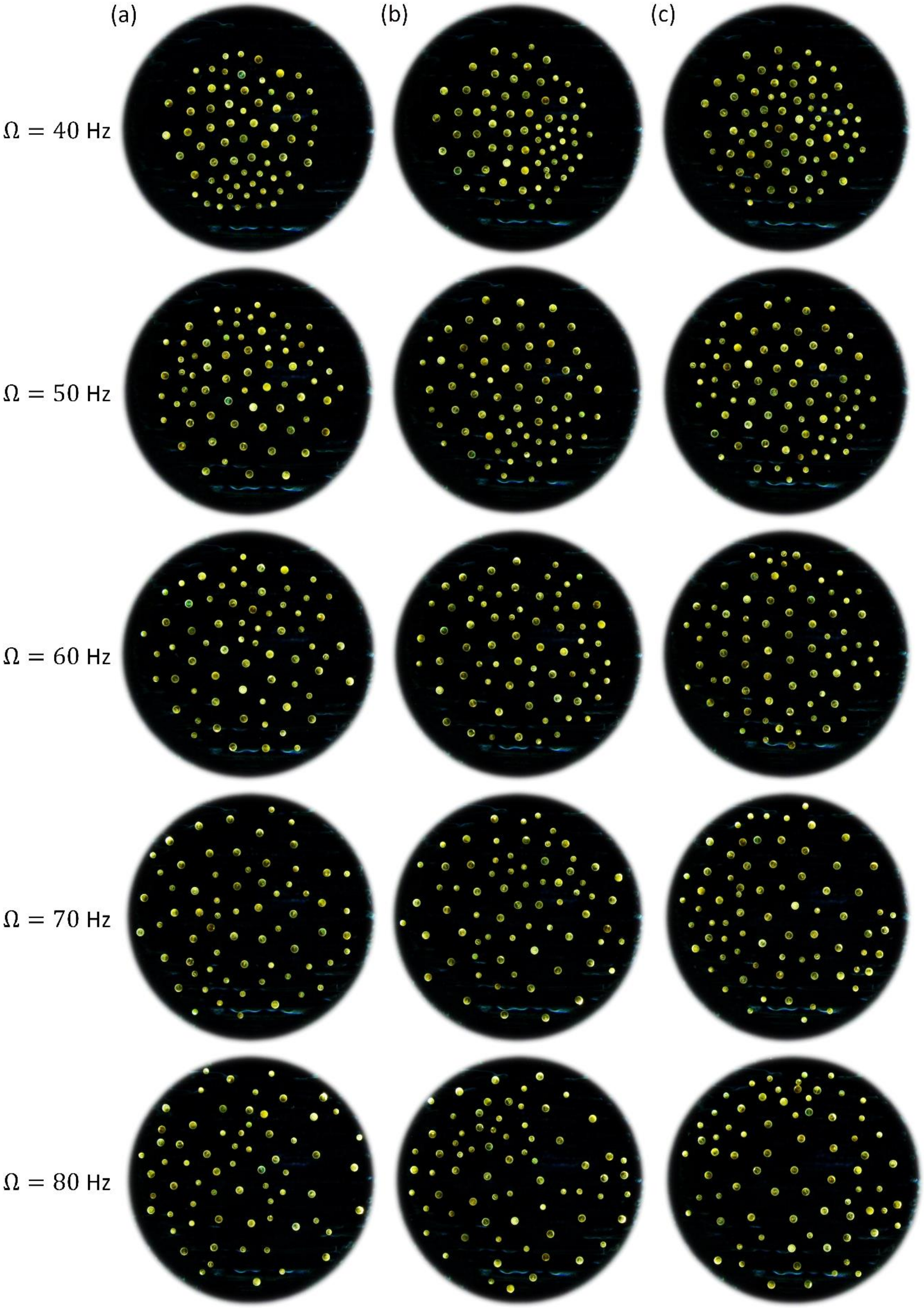

Fig. S4. Emergent self-organization of collectives with $\boldsymbol{R_1 = 200\ \mu}$m, $\boldsymbol{R_1 = 150\ \mu}$m. (a) $A_{12} = 1.6$. **(b)** $A_{12} = 1.8$. **(c)** $A_{12} = 2.0$.

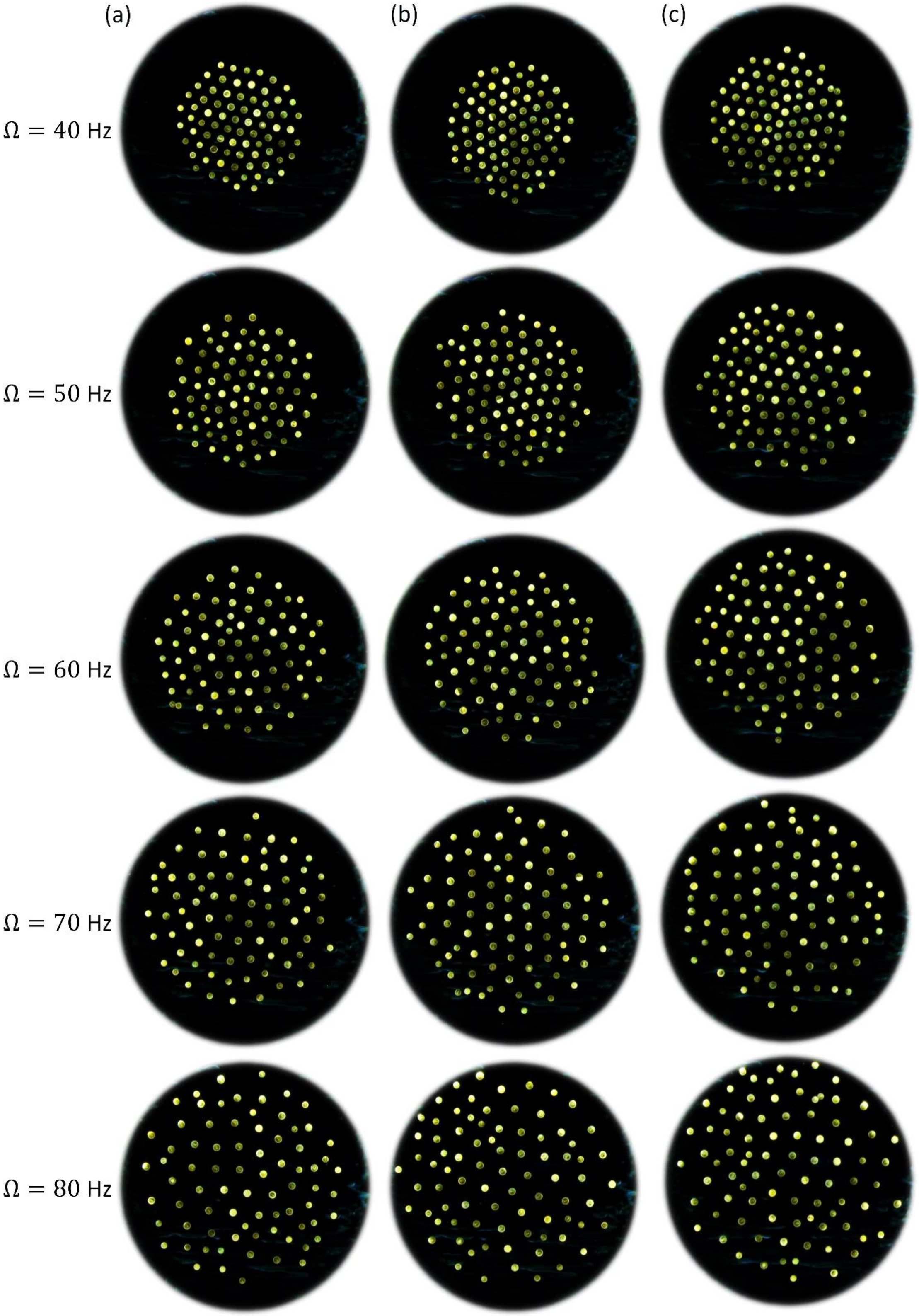

Fig. S5. Emergent self-organization of collectives with $\boldsymbol{R_1 = 175\ \mu}$m, $\boldsymbol{R_1 = 150\ \mu}$m. (a) $A_{12} = 1.6$. **(b)** $A_{12} = 1.8$. **(c)** $A_{12} = 2.0$.

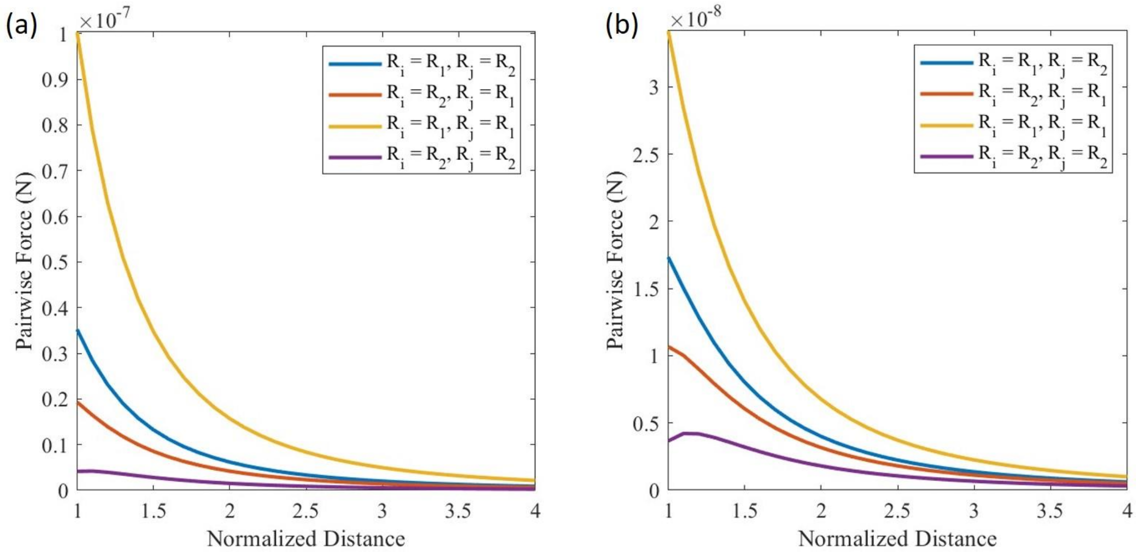

Fig. S6. Pairwise Interactions. (a-c) Pairwise forces between micro-disks of similar and different sizes at different distances when $\Omega = 40$ Hz. **(a)** Collectives with $R_1 = 200\ \mu$m and $R_2 = 150\ \mu$m. **(b)** Collectives with $R_1 = 175\ \mu$m and $R_2 = 150\ \mu$m. The distance is normalized by $(R_1 + R_2)/2$.

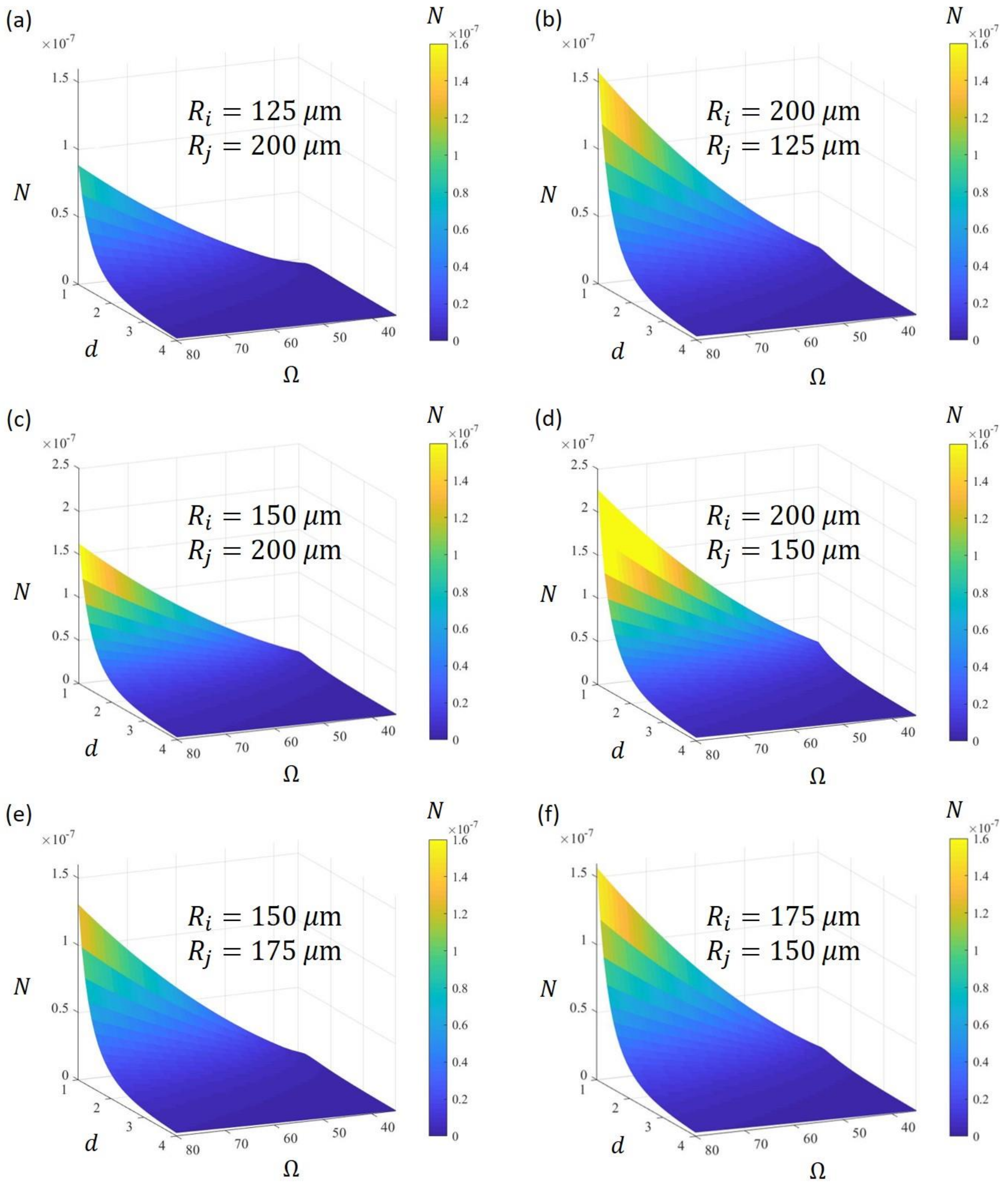

Fig. S7. Frequency-Dependent Pairwise Interactions 3D Maps. Pairwise forces between micro-disks of different sizes across the parameter space of interparticle distance (d) and magnetic field frequency (Ω) for collectives with $R_1 = 200\ \mu$m and $R_2 = 125\ \mu$m **(a-b)**, $R_1 = 200\ \mu$m and $R_2 = 150\ \mu$m **(c-d)**, $R_1 = 175\ \mu$m and $R_2 = 150\ \mu$m **(e-f)**. The distance d is normalized by $(R_1 + R_2)/2$.

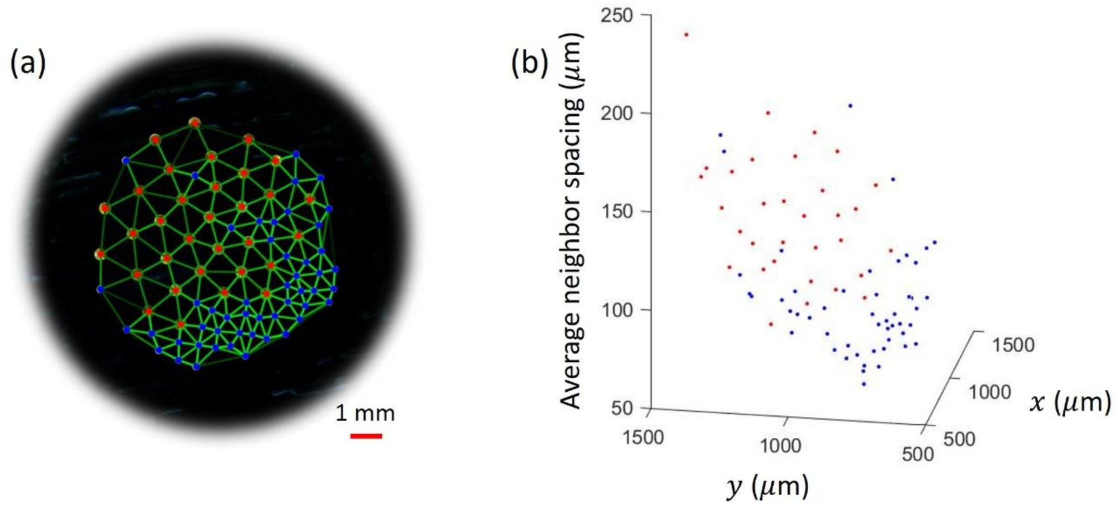

Fig. S8. Average neighbor distance 3D plot. (a) Heterogeneous collective with distance-weighted shading between neighboring micro-disks when $R_1 = 200\ \mu$m and $R_2 = 125\ \mu$m. **(b)** Corresponding 3D plot where each micro-disk is plotted by its x and y positions and according to its average neighbor distance on the z axis. Red dots correspond to micro-disks with a radius of R_1 and blue dots correspond to micro-disks with a radius of R_2.

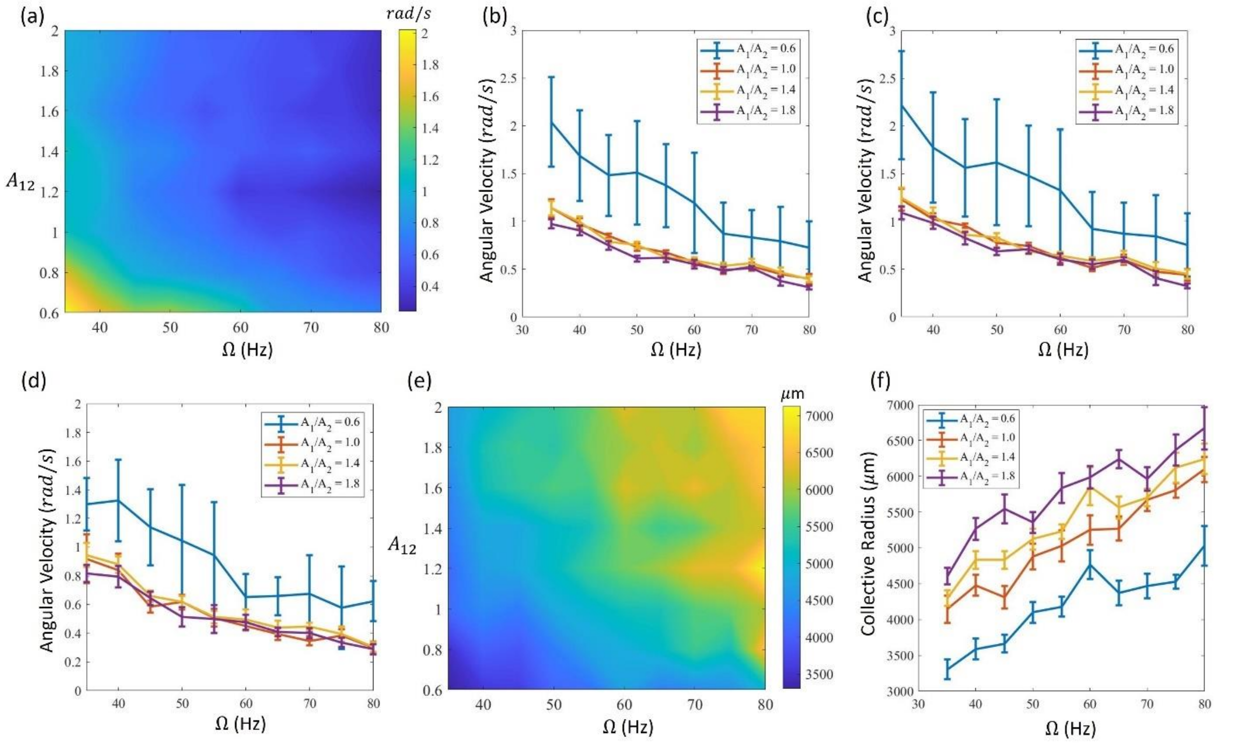

Fig. S9. Characterization of collectives with $R_1 = 200\ \mu$m, $R_1 = 125\ \mu$m. (a) Angular velocity across $A_{12} - \Omega$ parameter space. **(b-d)** Angular velocity line plots at $A_{12} \in [0.6, 1.0, 1.4, 1.8]$. **(b)** Collective's average angular velocity. **(c)** Average angular velocity of micro-disks with a radius of $125\ \mu$m. **(d)** Average angular velocity of micro-disks with a radius of $200\ \mu$m. **(e)** Collective radius across $A_{12} - \Omega$ parameter space. **(f)** Collective radius line plots at $A_{12} \in [0.6, 1.0, 1.4, 1.8]$.

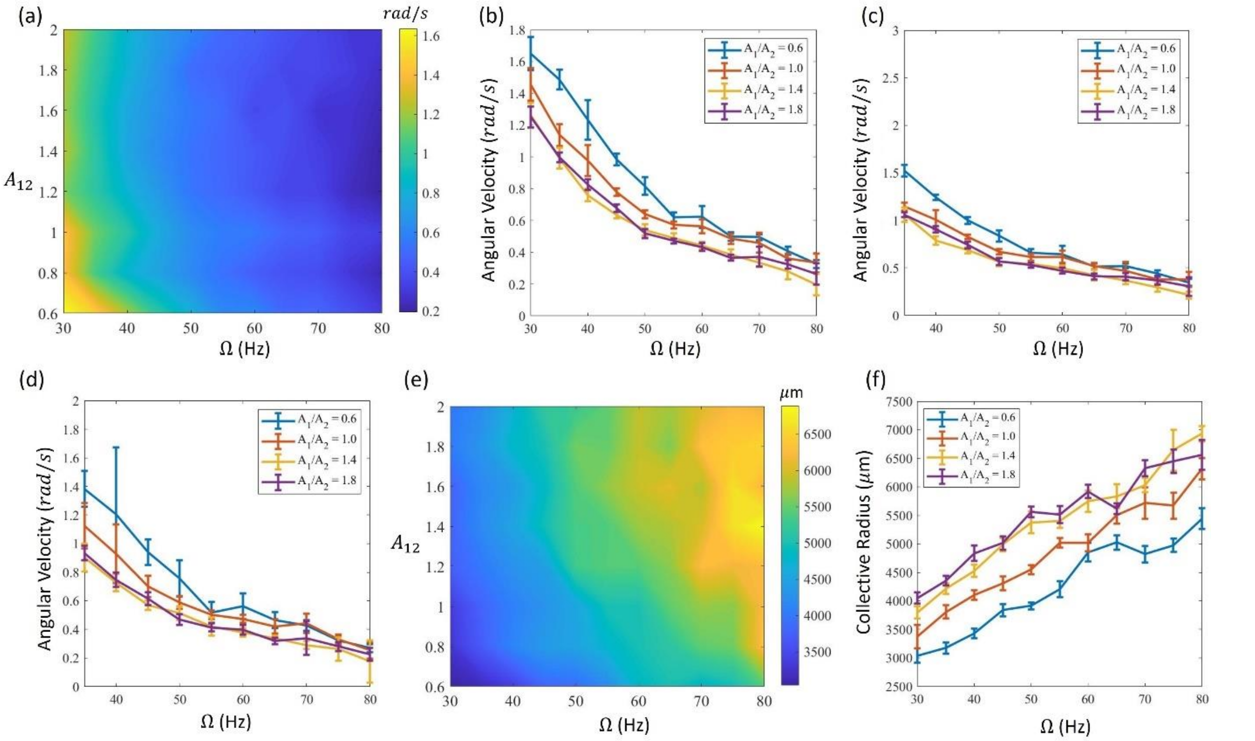

Fig. S10. Characterization of collectives with $R_1 = 200\ \mu$m, $R_1 = 150\ \mu$m. (a) Angular velocity across $A_{12} - \Omega$ parameter space. **(b-d)** Angular velocity line plots at $A_{12} \in [0.6, 1.0, 1.4, 1.8]$. **(b)** Collective's average angular velocity. **(c)** Average angular velocity of micro-disks with a radius of $150\ \mu$m. **(d)** Average angular velocity of micro-disks with a radius of $200\ \mu$m. **(e)** Collective radius across $A_{12} - \Omega$ parameter space. **(f)** Collective radius line plots at $A_{12} \in [0.6, 1.0, 1.4, 1.8]$.

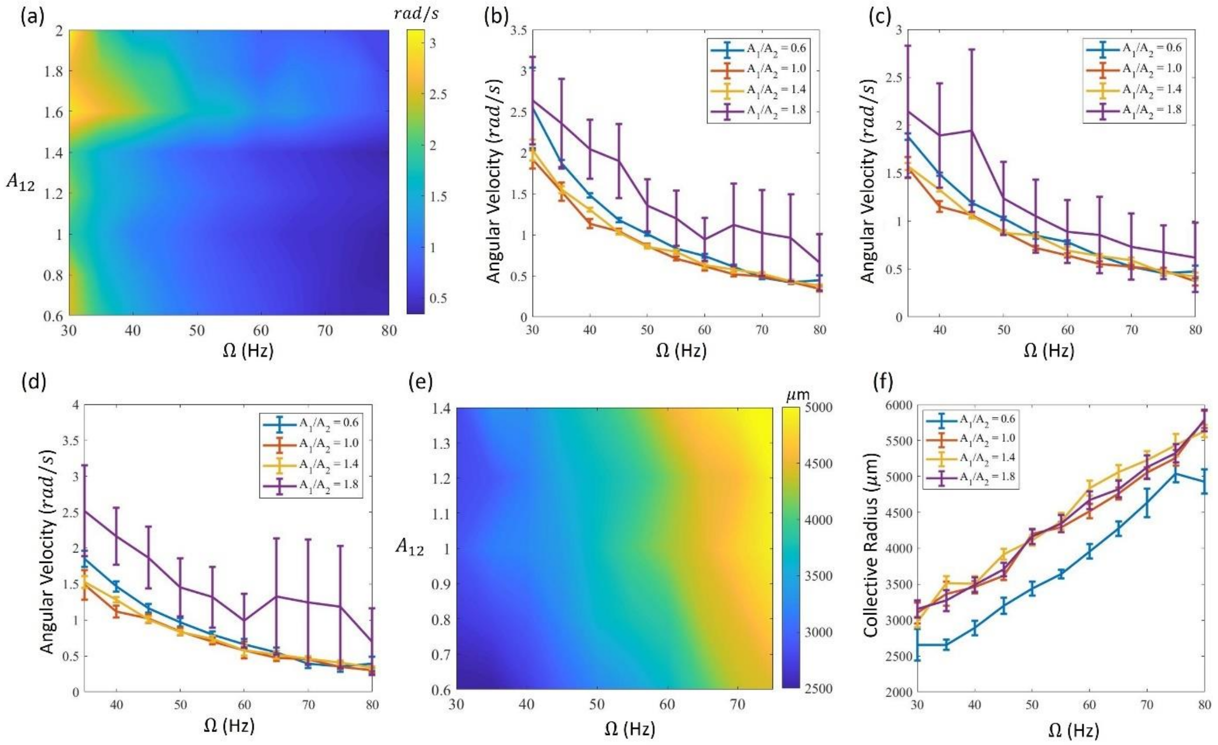

Fig. S11. Characterization of collectives with $R_1 = 175\ \mu$m, $R_1 = 150\ \mu$m. (a) Angular velocity across $A_{12} - \Omega$ parameter space. **(b-d)** Angular velocity line plots at $A_{12} \in [0.6, 1.0, 1.4, 1.8]$. **(b)** Collective's average angular velocity. **(c)** Average angular velocity of micro-disks with a radius of $150\ \mu$m. **(d)** Average angular velocity of micro-disks with a radius of $175\ \mu$m. **(e)** Collective radius across $A_{12} - \Omega$ parameter space. **(f)** Collective radius line plots at $A_{12} \in [0.6, 1.0, 1.4, 1.8]$.

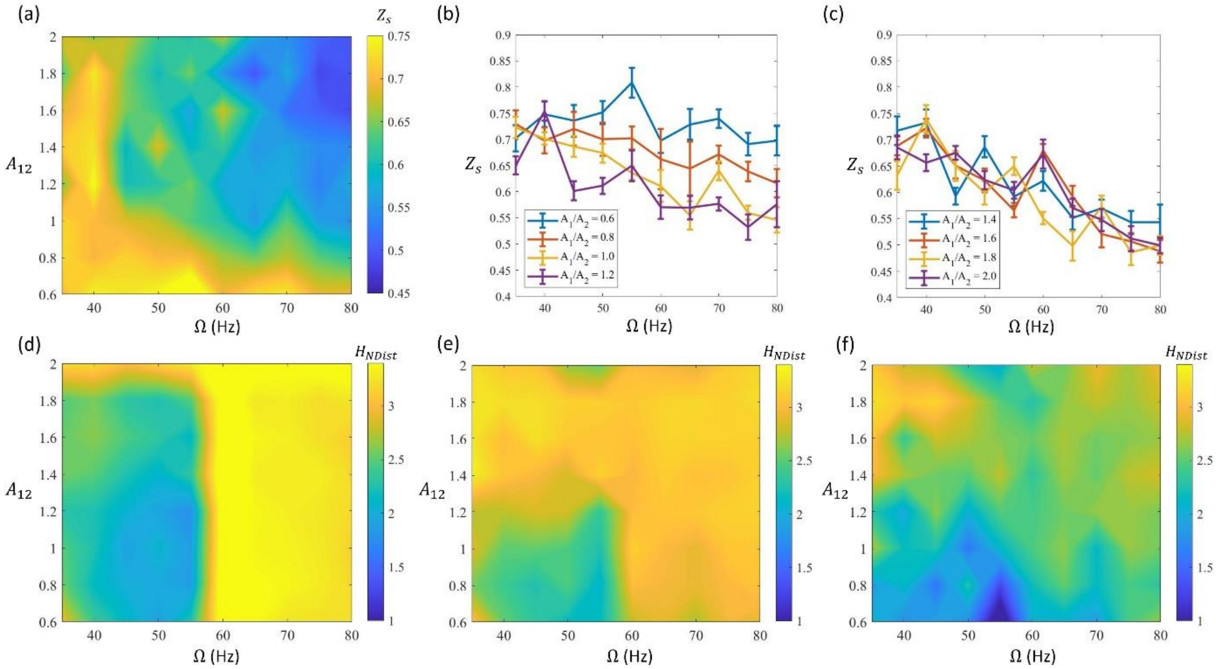

Fig. S12. Self-organization and entropy of collectives with $\boldsymbol{R_1 = 200\ \mu}$m, $\boldsymbol{R_1 = 125\ \mu}$m. (a) Separation order across $A_{12} - \Omega$ parameter space. **(b)** Separation order line plots at $A_{12} \in [0.6, 0.8, 1.0, 1.2]$. **(c)** Separation order line plots at $A_{12} \in [1.4, 1.6, 1.8, 2.0]$. **(d)** H_{NDist} for only micro-disks with a radius of $200\ \mu$m across $A_{12} - \Omega$ parameter space. **(e)** H_{NDist} for all micro-disks across $A_{12} - \Omega$ parameter space. **(f)** H_{NDist} for only micro-disks with a radius of $125\ \mu$m across $A_{12} - \Omega$ parameter space.

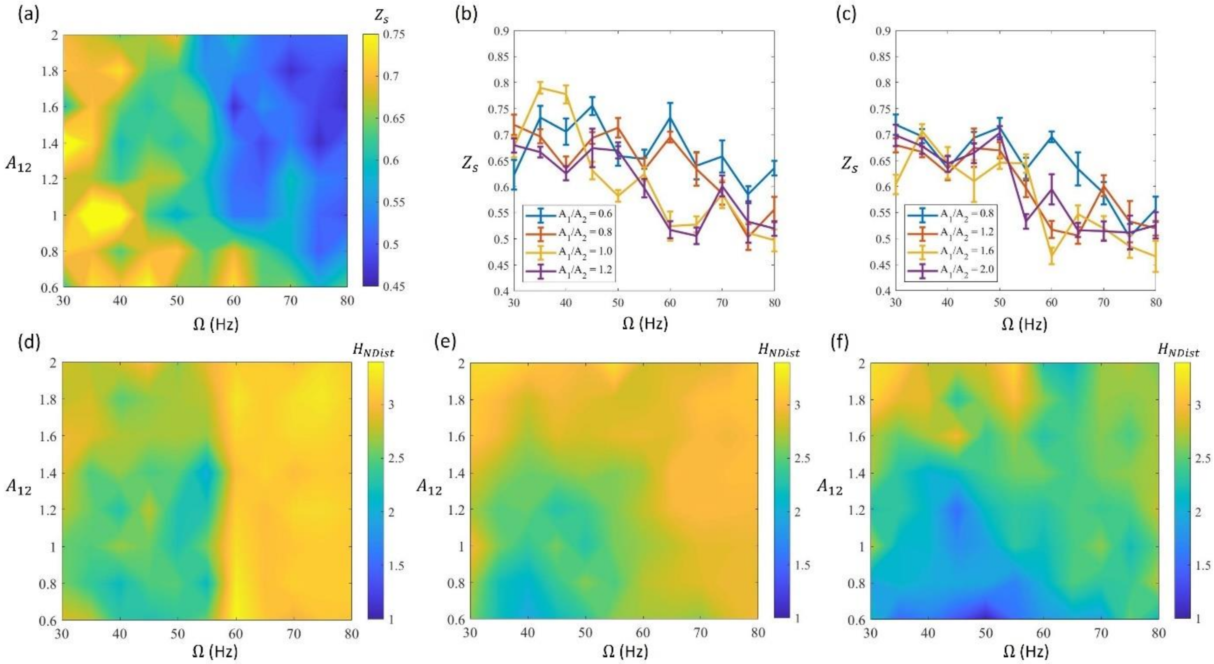

Fig. S13. Self-organization and entropy of collectives with $\boldsymbol{R_1 = 200\ \mu}$m, $\boldsymbol{R_1 = 150\ \mu}$m. (a) Separation order across $A_{12} - \Omega$ parameter space. **(b)** Separation order line plots at $A_{12} \in [0.6, 0.8, 1.0, 1.2]$. **(c)** Separation order line plots at $A_{12} \in [1.4, 1.6, 1.8, 2.0]$. **(d)** H_{NDist} for only micro-disks with a radius of $200\ \mu$m across $A_{12} - \Omega$ parameter space. **(e)** H_{NDist} for all micro-disks across $A_{12} - \Omega$ parameter space. **(f)** H_{NDist} for only micro-disks with a radius of $150\ \mu$m across $A_{12} - \Omega$ parameter space.

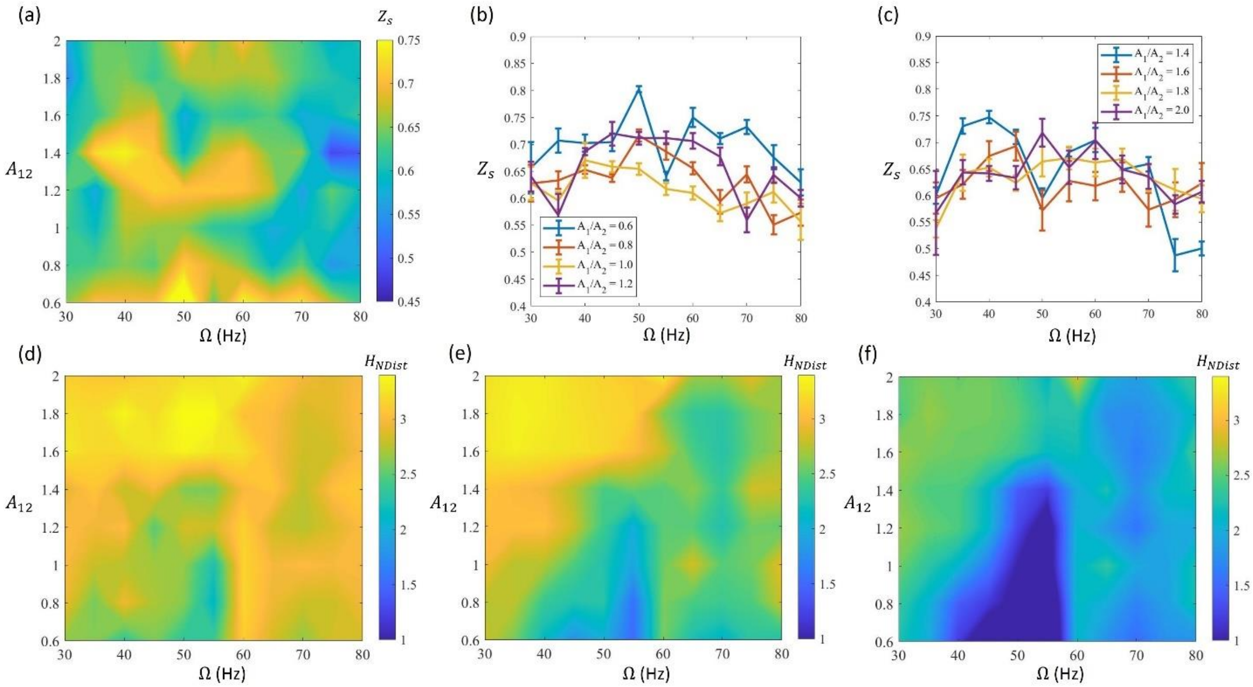

Fig. S14. Self-organization and entropy of collectives with $R_1 = 175\ \mu$m, $R_1 = 150\ \mu$m. (a) Separation order across $A_{12} - \Omega$ parameter space. **(b)** Separation order line plots at $A_{12} \in [0.6, 0.8, 1.0, 1.2]$. **(c)** Separation order line plots at $A_{12} \in [1.4, 1.6, 1.8, 2.0]$. **(d)** H_{NDist} for only micro-disks with a radius of $175\ \mu$m across $A_{12} - \Omega$ parameter space. **(e)** H_{NDist} for all micro-disks across $A_{12} - \Omega$ parameter space. **(f)** H_{NDist} for only micro-disks with a radius of $150\ \mu$m across $A_{12} - \Omega$ parameter space.

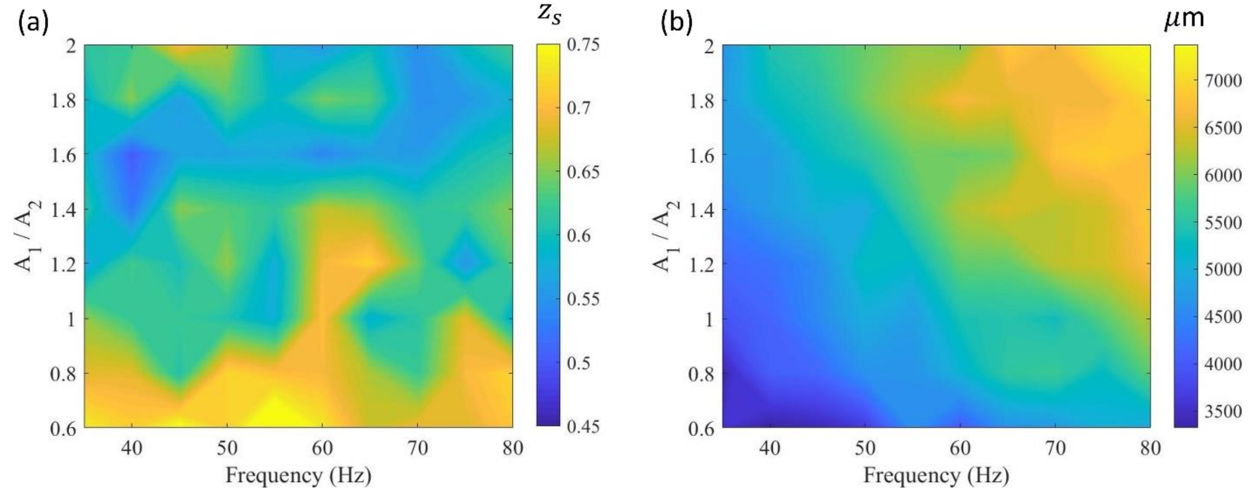

Fig. S15. Physical model characterization. (a) Heat map of the separation order parameter Z_s across the parameter space of magnetic field frequency and area ratio of micro-disks ($\Omega - A_{12}$) for $R_1 = 200\ \mu$m and $R_2 = 125\ \mu$m. **(b)** Heat map of collective radius.

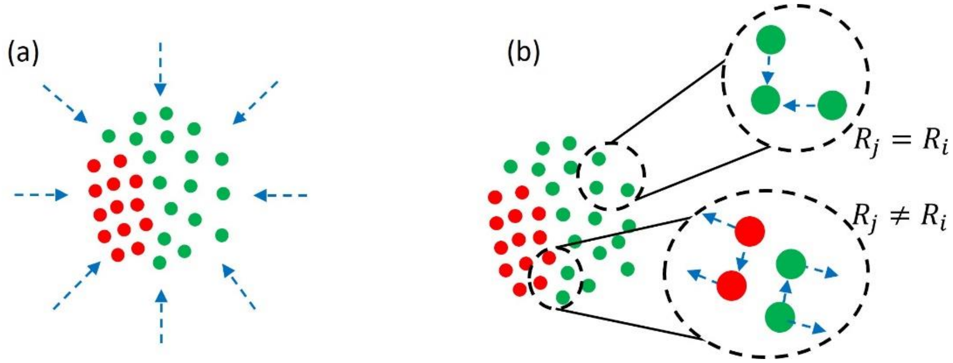

Fig. S16. Swarmalator model overview. (a) All micro-disks have an underlying attraction to each other that simulates the circular arena's confinement effect**. (b)** Micro-disks with differing radii tend to move away from each other while those with the same radii move toward each other.

Supplementary Movies

Movie S1. Homogeneous collective $\boldsymbol{R = 125\ \mu}$m. A homogeneous collective of micro-disks with $R = 200\ \mu$m rotates about its center when the $\Omega = 45$ Hz.
Movie S2. Heterogeneous Collectives Transition Between Order and Disorder. Heterogeneous collective with $R_1 = 200\ \mu$m and $R_1 = 125\ \mu$m transitions from a disordered mixture of particles of different sizes to an order separation by size.
Movie S3. Heterogeneous Collectives ($\boldsymbol{R_1 = 200\ \mu}$m, $\boldsymbol{R_2 = 125\ \mu}$m). Heterogeneous collectives demonstrating sustained separation at various frequencies and area ratios.
Movie S4. Heterogeneous Collectives ($\boldsymbol{R_1 = 200\ \mu}$m, $\boldsymbol{R_2 = 150\ \mu}$m). Heterogeneous collectives demonstrating sustained separation at various frequencies and area ratios.
Movie S5. Heterogeneous Collectives ($\boldsymbol{R_1 = 175\ \mu}$m, $\boldsymbol{R_2 = 150\ \mu}$m). Heterogeneous collectives demonstrating minimal separation at various frequencies and area ratios.
Movie S6. Heterogeneous Collectives ($\boldsymbol{R_1 = 200\ \mu}$m, $\boldsymbol{R_2 = 50\ \mu}$m) $\boldsymbol{N_{R_1} = 25, N_{R_2} = 100.}$ Heterogeneous collectives demonstrating separation at various frequencies.
Movie S7. Heterogeneous Collectives ($\boldsymbol{R_1 = 200\ \mu}$m, $\boldsymbol{R_2 = 125\ \mu}$m, $\boldsymbol{R_3 = 50\ \mu}$m). Heterogeneous collectives of three groups ($R_1 = 200\ \mu$m, $R_2 = 125\ \mu$m, $R_2 = 50\ \mu$m)
demonstrating partial separation at various frequencies.
Movie S8. Organized Static Aggregation. Heterogeneous collectives demonstrating sustained separation when the collective transitions to a static, aggregated state.
Movie S9. Flow Visualization. Visualization of flows throughout the collective when the collective is rotating and when it transitions to a static, aggregated state.
Movie S10. Organized Dispersal at 100 Hz. Heterogeneous collectives partially beyond their step out frequency and exhibiting an organized form of dispersal in which large micro-disks move toward the boundary.
Movie S11. Physical Model Simulations of Heterogeneous Collectives ($\boldsymbol{R_1 = 200\ \mu}$m, $\boldsymbol{R_2 = 125\ \mu}$m). Simulation shows a collective with $R_1 = 200\ \mu$m and $R_1 = 125\ \mu$m transitioning from disorder to order.
Movie S12. Swarmalator Model Simulations of Heterogeneous Collectives ($\boldsymbol{R_1 = 200\ \mu}$m, $\boldsymbol{R_2 = 125\ \mu}$m). Swarmalator collectives transitioning from a disordered mixture to ordered mixture at 40 and 80 Hz.
Movie S13. Anisotropic Deformation Under Isotropic Compression. Self-organized collective deforms into an ellipse-like configuration after being compressed isotropically.
Movie S14. Organized Collective Locomotion. A self-organized collective maintains its organized state as it moves.
Movie S15. Caging and Expulsion of Passive Objects. Passive objects of different sizes are caged and expelled according to their size.
